# Disparities in accelerated brain aging in recent-onset and chronic schizophrenia

**DOI:** 10.1017/S0033291725000285

**Published:** 2025-02-24

**Authors:** Sung Woo Joo, Junhyeok Lee, Juhyuk Han, Minjae Kim, Yeonwoo Kim, Howook Lee, Young Tak Jo, Jaewook Shin, Jungsun Lee, Won Hee Lee

**Affiliations:** 1 Department of Psychiatry, Asan Medical Center, University of Ulsan College of Medicine, Seoul, Republic of Korea; 2 Department of Software Convergence, Kyung Hee University, Yongin, Republic of Korea; 3 Department of Artificial Intelligence, Kyung Hee University, Yongin, Republic of Korea; 4 Department of Psychiatry, Kangdong Sacred Heart Hospital, Seoul, Republic of Korea; 5 Department of Medicine, CHA University School of Medicine, Seongnam, Republic of Korea

**Keywords:** brain aging, schizophrenia, recent-onset, chronic

## Abstract

**Background:**

Patients with schizophrenia experience accelerated aging, accompanied by abnormalities in biomarkers such as shorter telomere length. Brain age prediction using neuroimaging data has gained attention in schizophrenia research, with consistently reported increases in brain-predicted age difference (brain-PAD). However, its associations with clinical symptoms and illness duration remain unclear.

**Methods:**

We developed brain age prediction models using structural magnetic resonance imaging (MRI) data from 10,938 healthy individuals. The models were validated on an independent test dataset comprising 79 healthy controls, 57 patients with recent-onset schizophrenia, and 71 patients with chronic schizophrenia. Group comparisons and the clinical associations of brain-PAD were analyzed using multiple linear regression. SHapley Additive exPlanations (SHAP) values estimated feature contributions to the model, and between-group differences in SHAP values and group-by-SHAP value interactions were also examined.

**Results:**

Patients with recent-onset schizophrenia and chronic schizophrenia exhibited increased brain-PAD values of 1.2 and 0.9 years, respectively. Between-group differences in SHAP values were identified in the right lateral prefrontal area (false discovery rate [FDR] p = 0.022), with group-by-SHAP value interactions observed in the left prefrontal area (FDR p = 0.049). A negative association between brain-PAD and Full-scale Intelligence Quotient scores in chronic schizophrenia was noted, which did not remain significant after correction for multiple comparisons.

**Conclusions:**

Brain-PAD increases were pronounced in the early phase of schizophrenia. Regional brain abnormalities contributing to brain-PAD likely vary with illness duration. Future longitudinal studies are required to overcome limitations related to sample size, heterogeneity, and the cross-sectional design of this study.

## Introduction

Numerous studies have reported that individuals with schizophrenia have a life expectancy reduced by approximately 10–25 years, primarily due to an increased risk of suicide and comorbidities such as dyslipidemia, diabetes mellitus, and metabolic syndrome (Correll et al., 2022; Jayatilleke et al., 2017; Tanskanen, Tiihonen, & Taipale, 2018). Abnormalities in various biomarkers associated with the aging process, such as shorter telomere length (Russo et al., 2018), oxidative stress (Flatow, Buckley, & Miller, 2013), and higher inflammatory markers (Lee, Hong, Martin, Eyler, & Jeste, 2017), have also been observed in patients with schizophrenia. According to the findings, patients with schizophrenia may undergo accelerated aging and premature death compared to healthy individuals (Nguyen, Eyler, & Jeste, 2017). As aging affects the brain, cognitive functions, which are associated with brain aging, are often impaired before or at the onset of psychotic symptoms in individuals with schizophrenia (McCutcheon, Keefe, & McGuire, 2023). Extensive evidence on structural brain abnormalities in schizophrenia further supports the notion of an accelerated aging process in the brains of patients with schizophrenia (Constantinides et al., 2023).

Brain age prediction using neuroimaging data has garnered significant attention in schizophrenia research, with more than 20 studies being published on this topic. Brain-predicted age difference (brain-PAD), calculated as the difference between neuroimaging-based brain age and chronological age, is increased by 3.5 years in patients with schizophrenia compared with healthy individuals (Constantinides et al., 2023). This increase in brain aging in patients with schizophrenia is more pronounced in the early phase of the illness, particularly within the first 5 years of onset than in the later phases. Schnack et al., in a longitudinal study, reported that the brain-PAD in schizophrenia persisted over the follow-up period from baseline, although the extensive variability in brain abnormalities during follow-up rendered the increased gap statistically non-significant (Hugo G Schnack et al., 2016). In terms of brain-PAD changes across different illness stages, Kim et al. reported that patients with treatment-resistant schizophrenia exhibited a more substantial brain age acceleration compared to those with first-episode schizophrenia spectrum disorder (Kim et al., 2024; Kim et al., 2023). While existing research consistently indicates increased brain-PAD in schizophrenia, the relationship between brain-PAD and illness duration remains unclear.

The clinical implications of elevated brain-PAD in patients with schizophrenia remain uncertain. Two large-scale studies have found no significant associations between brain-PAD and various clinical factors, including the severity of psychiatric symptoms, antipsychotic dosage, and illness duration (Constantinides et al., 2023; Kaufmann et al., 2019). Previous studies consistently report an increased brain-PAD in patients with schizophrenia; however, the inter-subject variability in brain-PAD remains unresolved and warrants future research. The clinical heterogeneity of schizophrenia is well-recognized, particularly in terms of response to antipsychotic treatment, long-term clinical course, and prognosis (Bosia et al., 2019; Buchanan & Carpenter, 1994; Dickinson et al., 2018). The first five years following the onset of schizophrenia are widely recognized as a critical period that influences long-term clinical outcomes and prognosis (Birchwood, Todd, & Jackson, 1998). Numerous studies have reported that beyond this early phase, patients with schizophrenia often demonstrate reduced responsiveness to antipsychotic treatment and encounter greater challenges in regaining premorbid functioning (McGorry, Hickie, Yung, Pantelis, & Jackson, 2006). Considering this clinical trajectory, categorizing patients into recent-onset and chronic groups is advantageous, as these groups are likely to exhibit distinct neurobiological characteristics (Wood, Yung, McGorry, & Pantelis, 2011). Although definitions of “recent-onset” and “chronic” schizophrenia vary across studies, the first five years are frequently adopted as a benchmark for defining recent-onset schizophrenia (Newton et al., 2018).

Brain age prediction, which estimates an individual’s biological brain age based on neuroimaging data, has emerged as a valuable tool for understanding brain development and aging. However, traditional approaches for brain age prediction have several limitations. Previous studies have predominantly relied on single-model approaches, which may not fully capture the complexity and diversity of brain structures and functions (Franke, Ziegler, Klöppel, Gaser, & Initiative, 2010). Additionally, many studies have been constrained by small sample sizes, limiting the generalizability and predictive accuracy of their models (Conrad, Mälzer, Schwarzenberger, Wiemer, & Ihlenfeldt, 2022; Safonova et al., 2023). Multiple studies have demonstrated that smaller datasets significantly impact model performance, highlighting the critical need for larger datasets to enhance prediction accuracy and generalizability (Cole et al., 2017). Ensemble learning methods address these challenges by combining predictions from multiple models, thereby compensating for individual model weaknesses and enhancing predictive stability and accuracy (Kyriakides & Margaritis, 2019). This approach is particularly valuable in brain age prediction, where diverse brain characteristics must be comprehensively considered (Couvy-Duchesne et al., 2020; Kuo et al., 2021; Xiong et al., 2023). Furthermore, utilizing large datasets can significantly improve the performance of machine learning models in brain age prediction. Large datasets that encompass diverse populations and brain structures enable models to learn from a wider range of cases, thereby improving their ability to generalize. These expanded datasets allow models to capture infrequent changes in brain structure or function that can be crucial for accurate age prediction.

**Figure 1.**
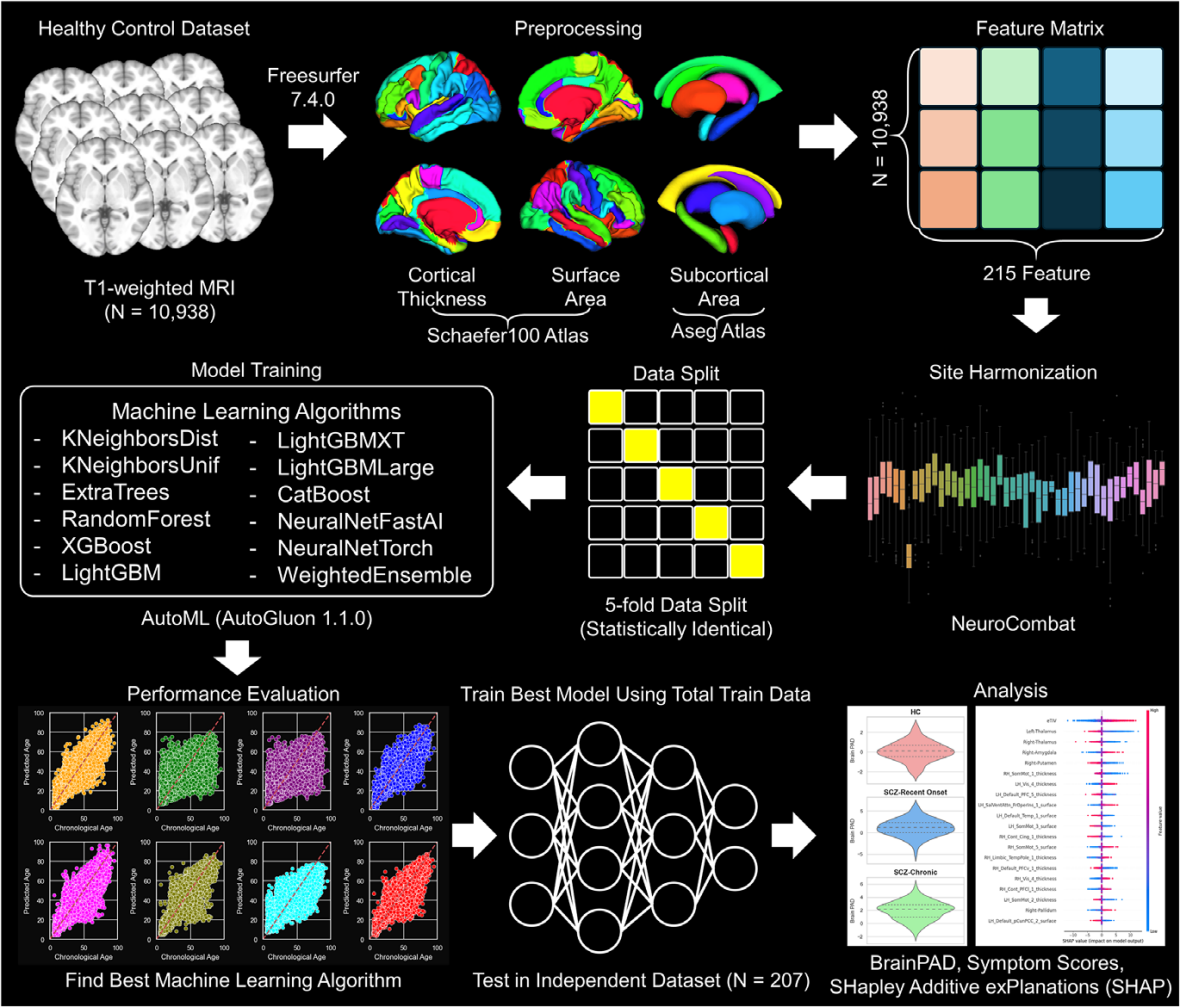
Overview of the workflow for brain age prediction.

In this study, we used neuroimaging data from over 10,000 healthy individuals, sourced from 20 public databases, to develop brain age prediction models. These models were validated on independent test data of healthy controls and patients with schizophrenia. The patients with schizophrenia were categorized into the recent-onset and chronic schizophrenia groups based on an illness duration of 5 years. We analyzed changes in brain-PAD across these groups and further explored the associations between brain-PAD and clinical variables. Figure 1 illustrates the workflow for brain age prediction in our study.

## Methods

### Study samples

To develop a brain age prediction model, we collected de-identified T1-weighted MRI data from 20 public databases. The final dataset comprised 10,938 healthy individuals aged between 5 and 95 years. Detailed demographics and dataset-specific characteristics are provided in Supplementary Table 1.

For the independent test sample, we combined data from three different cohorts recruited from the Asan Medical Center, a university-affiliated hospital. The first cohort (AMC 1) included 49 patients with recent-onset schizophrenia and 24 healthy controls. The second cohort (AMC 2) comprised 27 patients with schizophrenia, 24 patients with bipolar disorder, and 55 healthy controls. The third cohort (AMC 3) involved 52 patients with chronic schizophrenia. We excluded three participants who enrolled in more than one of these cohorts. Detailed information about each cohort can be found in the Supplementary Material. We categorized patients with schizophrenia as having recent-onset schizophrenia or chronic schizophrenia based on an illness duration of 5 years (Newton et al., 2018). Illness duration was defined as the period from the onset of psychotic symptoms to the date of the clinical interview with participants. After excluding patients with bipolar disorders, the independent test sample included 79 healthy controls, 57 patients with recent-onset schizophrenia, and 71 patients with chronic schizophrenia. Different tools were employed across cohorts to assess neurocognitive performance and psychiatric symptoms. The severity of psychiatric symptoms was measured using the Positive and Negative Syndrome Scale (PANSS) (Kay, Fiszbein, & Opler, 1987) for patients in the AMC 1 and AMC 3 cohorts. Full-scale Intelligence Quotient (FSIQ) and Memory Quotient (MQ) scores were also collected for participants in the AMC 1 and AMC 3 cohorts. Participants in the AMC 2 cohort were assessed using the Cogstate brief battery (Maruff et al., 2009), which solely focuses on cognitive functions. Further details on the clinical and cognitive assessments used in each cohort are provided in the Supplementary Material.

The authors assert that all procedures contributing to this work comply with the ethical standards of the relevant national and institutional committees on human experimentation and with the Helsinki Declaration of 1975, as revised in 2008. The present study was approved by the IRB of the Asan Medical Center (IRB No. 2021–1128).

### Image acquisition, preprocessing, and analysis

Information on scanners and T1-weighted MRI acquisition parameters for the training samples and test samples are detailed in Supplementary Tables 2 and 3. All MRI data were rigorously inspected to ensure quality, and only participants with MRI data deemed adequate for downstream analysis were included in the final study population. T1-weighted images from all participants were processed identically using the automated FreeSurfer v7.4 (http://surfer.nmr.mgh.harvard.edu) pipeline. For cortical parcellation, we utilized the Schaefer atlas (Schaefer et al., 2018), chosen for its capacity to parcellate the cerebral cortex based on intrinsic functional connectivity MRI, enabling a higher degree of functional integration and segregation, thus yielding neurobiologically meaningful features. Additionally, we selected seven subcortical regions from each hemisphere—accumbens, amygdala, caudate, hippocampus, thalamus, pallidum, and putamen. This resulted in 215 brain features, including cortical thickness and surface area of 100 cortical regions, volumes of 14 subcortical regions, and total intracranial volume. To account for site-specific variability inherent in this multi-center neuroimaging study (Dufumier et al., 2022), we applied neuroCombat (Fortin et al., 2018), an adaptation of the ComBat harmonization method (Johnson, Li, & Rabinovic, 2007), which effectively mitigates site effects. Details on the effectiveness of ComBat harmonization are provided in the Supplementary Material.

### Model development and validation

We trained and evaluated 32 models, including base models and their ensemble variants, which utilized stacking and bagging techniques to enhance predictive performance. Detailed descriptions for each algorithm and stacking and bagging techniques are described in Supplementary Material.

### Model training

The brain age prediction model was trained using AutoGluon (Erickson et al., 2020), an open-source automated machine learning (AutoML) library chosen for its efficiency and ease of use. AutoGluon supports high-level tuning, stacking, and bagging ensemble techniques with minimal code using preset configurations. We used the “best_quality” preset, which, although computationally intensive, applies advanced techniques to optimize performance. For bagging, we employed 8-fold cross-validation to enhance model stability and generalization, allowing the system to integrate the strengths of diverse models for potentially superior prediction accuracy.

Following comprehensive performance evaluations, we selected the best-performing model to conduct further analyses on brain age prediction. To assess model reliability, we performed 5-fold cross-validation within the training dataset, identifying the model with the highest performance, which was then retrained on all available training samples to maximize predictive power. This retrained model, optimized on the full dataset, was used for the final analysis.

Model training was optimized using root mean squared error (RMSE) as the loss function to enhance prediction accuracy. The training process was conducted on a hardware setup comprising an Intel(R) Core(TM) i9-10900X CPU @ 3.70GHz and four NVIDIA RTX 4090 24GB GPUs, running on Ubuntu 20.04.6 LTS.

### Statistical analysis

All statistical analyses were conducted using R software (version 4.0.2; R Development Core Team, Vienna, Austria). Statistical significance was set at an alpha value of <0.05. Between-group differences in continuous variables were tested using the t-test or analysis of variance (ANOVA), as appropriate. The chi-square tests were applied to evaluate between-group differences in categorical variables.

Given the strong associations between chronological age and brain-PAD (Supplementary Figure 4), linear regression models were used to adjust brain-PAD for age, age-squared, and sex. We excluded outliers identified as values greater than 1.5 IQR above the median or lower than 1.5 IQR below the median of the age-adjusted brain-PAD values. Between-group comparisons of brain-PAD were performed using analysis of covariance (ANCOVA) with age, age-squared, and sex as covariates, and post-hoc analyses were conducted using Tukey’s method. Clinical associations with brain-PAD were examined using linear regression models, where unadjusted brain-PAD was the dependent variable, and clinical variables, age, age-squared, and sex were the independent variables. We conducted linear regression analyses within each group and determined the clinical associations based on the standardized coefficients for each clinical variable. Multiple comparisons were corrected using an FDR of q < 0.05, accounting for the number of clinical variables (n = 7).

Feature importance was assessed using SHapley Additive exPlanations (SHAP) values (Lundberg, 2017). SHAP values measure how much each feature contributes to a model’s predictions. By analyzing how predictions change when features are included or excluded, SHAP calculates the importance of each feature while accounting for their interactions, providing a fair assessment of feature impact. To derive feature importance, SHAP values were calculated for the entire test dataset, and the top 20 features were selected based on their absolute SHAP values. An ANOVA was used to examine between-group differences in SHAP values and group-by-SHAP interactions for the age-adjusted brain-PAD. Multiple comparisons were adjusted for the number of features (n = 20) using an FDR of q < 0.05.

## Results

### Demographic and clinical characteristics of the independent test sample

Table 1 presents the demographics and clinical characteristics of the participants in the independent test sample. Significant differences were observed among the three groups in age (F = 18.71, p < 0.001), FSIQ sores (F = 32.21, p < 0.001), and MQ scores (F = 31.33, p < 0.001). The mean illness durations in the recent-onset and chronic schizophrenia groups were 2.3 (3.7) and 15.1 (8.8) years, respectively. No significant differences in PANSS total and subscale scores were observed between the two patient groups. However, patients with chronic schizophrenia had significantly higher Global Assessment of Functioning scores than those with recent-onset schizophrenia (t = 6.428, p < 0.001).

**Table 1.** Demographics and clinical characteristics of the independent test sample

	Group			Statistic		
	HC	Recent-onset SCZ	Chronic SCZ	F or t	p	Post-hoc
Number of participants	79	57	71			
Age, mean (SD), year	32.6 (6.9)	28.5 (6.3)	37.7 (11.4)	18.71	<0.001	C > H > R
Male sex, n (%)	25 (31.7)	23 (40.4)	31(43.7)	2.447	0.294	
Duration of illness, mean (SD), year	NA	2.3 (3.7)	15.1 (8.8)	9.028	<0.001	
FSIQ, mean (SD)	120.1 (9.2)	97.2 (15.9)	90.4 (16.4)	32.21	<0.001	H > R, C
MQ, mean (SD)	109.4 (11.9)	83.8 (21.0)	73.1 (16.1)	31.33	<0.001	H > R > C
PANSS positive, mean (SD)	NA	16.6 (7.4)	14.5 (4.9)	−1.669	0.098	
PANSS negative, mean (SD)	NA	16.7 (7.1)	18.6 (6.5)	1.381	0.171	
PANSS general, mean (SD)	NA	28.9 (7.5)	32.5 (10.4)	1.979	0.051	
PANSS total, mean (SD)	NA	62.2 (15.9)	65.6 (19.7)	0.948	0.346	
GAF, mean (SD)	NA	44.1 (14.3)	63.1 (11.4)	6.428	<0.001	

C, chronic schizophrenia; FSIQ, Full-scale Intelligence Quotient; GAF, Global Assessment of Functioning; H, healthy controls; HC, healthy controls; MQ, Memory Quotient; NA, not available; PANSS, Positive and Negative Syndrome Scale; R, recent-onset schizophrenia; SCZ, schizophrenia.

### Model performance

We evaluated the performance of multiple machine learning models for brain age prediction using 5-fold cross-validation. Table 2 presents the performance metrics for each model, including mean absolute error (MAE), RMSE, Pearson’s correlation coefficient (R), and coefficient of determination (R^2^). Among the 32 models evaluated, the WeightedEnsemble_L3 model demonstrated the best performance, achieving the lowest MAE of 6.555 years. This model also recorded a strong R of 0.868 and R^2^ of 0.752.

**Table 2.** Performance evaluation of brain age prediction

Model	MAE	RMSE	R	R^2^
CatBoost_BAG_L1	7.518	10.173	0.848	0.718
CatBoost_BAG_L2	6.748	9.514	0.868	0.753
CatBoost_r177_BAG_L1	7.621	10.268	0.844	0.712
CatBoost_r177_BAG_L2	6.751	9.513	0.868	0.753
CatBoost_r9_BAG_L2	6.718	9.472	0.869	0.755
ExtraTreesMSE_BAG_L1	9.265	12.255	0.776	0.590
ExtraTreesMSE_BAG_L2	6.810	9.566	0.866	0.750
KNeighborsDist_BAG_L1	12.783	17.049	0.487	0.207
KNeighborsUnif_BAG_L1	12.810	17.071	0.485	0.205
LightGBMLarge_BAG_L1	7.949	10.770	0.830	0.684
LightGBMLarge_BAG_L2	6.674	9.477	0.869	0.755
LightGBMXT_BAG_L1	7.447	10.100	0.850	0.722
LightGBMXT_BAG_L2	6.731	9.482	0.869	0.755
LightGBM_BAG_L1	7.603	10.320	0.844	0.709
LightGBM_BAG_L2	6.717	9.500	0.868	0.754
LightGBM_r131_BAG_L2	6.699	9.482	0.869	0.755
LightGBM_r96_BAG_L2	6.751	9.493	0.868	0.754
NeuralNetFastAI_BAG_L1	7.012	9.819	0.859	0.737
NeuralNetFastAI_BAG_L2	7.013	9.713	0.862	0.743
NeuralNetFastAI_r191_BAG_L2	6.834	9.652	0.864	0.746
NeuralNetTorch_BAG_L1	6.812	9.928	0.856	0.731
NeuralNetTorch_BAG_L2	6.744	9.777	0.861	0.739
NeuralNetTorch_r22_BAG_L2	6.638	9.765	0.862	0.740
NeuralNetTorch_r79_BAG_L1	6.886	10.022	0.854	0.726
NeuralNetTorch_r79_BAG_L2	6.560	9.657	0.865	0.746
RandomForestMSE_BAG_L1	9.020	12.085	0.780	0.602
RandomForestMSE_BAG_L2	6.816	9.603	0.865	0.748
WeightedEnsemble_L2	6.732	9.635	0.864	0.747
WeightedEnsemble_L3	6.555	9.537	0.868	0.752
XGBoost_BAG_L1	7.782	10.528	0.837	0.698
XGBoost_BAG_L2	6.723	9.523	0.868	0.753
XGBoost_r33_BAG_L2	10.565	13.288	0.821	0.518

MAE, mean absolute error; R, Pearson’s correlation coefficient; R^2^, coefficient of determination; RMSE, root mean square error; L1, stacking level 1; L2, stacking level 2; L3, stacking level 3; BAG: bagging; r#, version identifier.

### Between-group differences in brain-PAD

**Figure 2.**
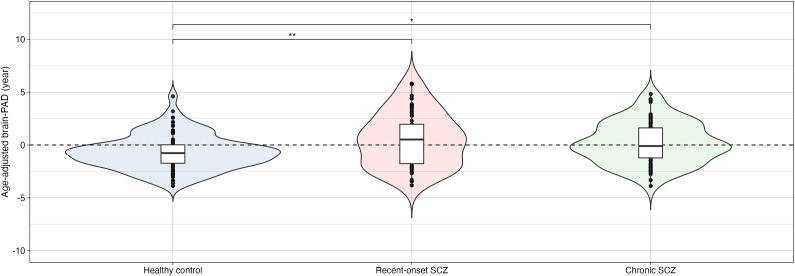
Between-group differences in age-adjusted brain-PAD among healthy controls, patients with recent-onset schizophrenia, and patients with chronic schizophrenia. Blue, pink, and green violin plots indicate healthy controls, patients with recent-onset schizophrenia, and patients with chronic schizophrenia. Brain-PAD, brain-predicted age difference; SCZ, schizophrenia; * p < 0.05, ** p < 0.01.

We performed an ANCOVA, adjusting for age, age-squared, and sex, to investigate between-group differences in brain PAD among healthy controls, patients with recent-onset schizophrenia, and patients with chronic schizophrenia. Figure 2 shows a significant between-group difference in brain-PAD (F = 281.086, p < 0.001). Compared with healthy controls, patients with recent-onset schizophrenia (t = 3.209, p = 0.004) and those with chronic schizophrenia (t = 2.742, p = 0.018) had greater brain-PADs. The mean age-adjusted brain-PADs of healthy controls, patients with recent-onset schizophrenia, and patients with chronic schizophrenia were − 0.70 (1.63), 0.48 (2.54), and 0.19 (1.96) years, respectively. Patients with recent-onset schizophrenia and those with chronic schizophrenia showed increased brain-PAD of 1.2 and 0.9 years, respectively, compared to healthy controls.

### Clinical association of brain-PAD

We performed linear regressions with covariates, including age and age-squared, to explore associations between brain-PAD and clinical symptoms (Supplementary Table 5). In patients with chronic schizophrenia, FSIQ scores demonstrated a negative association with brain-PAD (ß = −0.0402, uncorrected p = 0.042). However, after adjusting for multiple comparisons, this association was not significant.

### Between-group differences in SHAP values and group-by-SHAP value interactions

**Figure 3.**
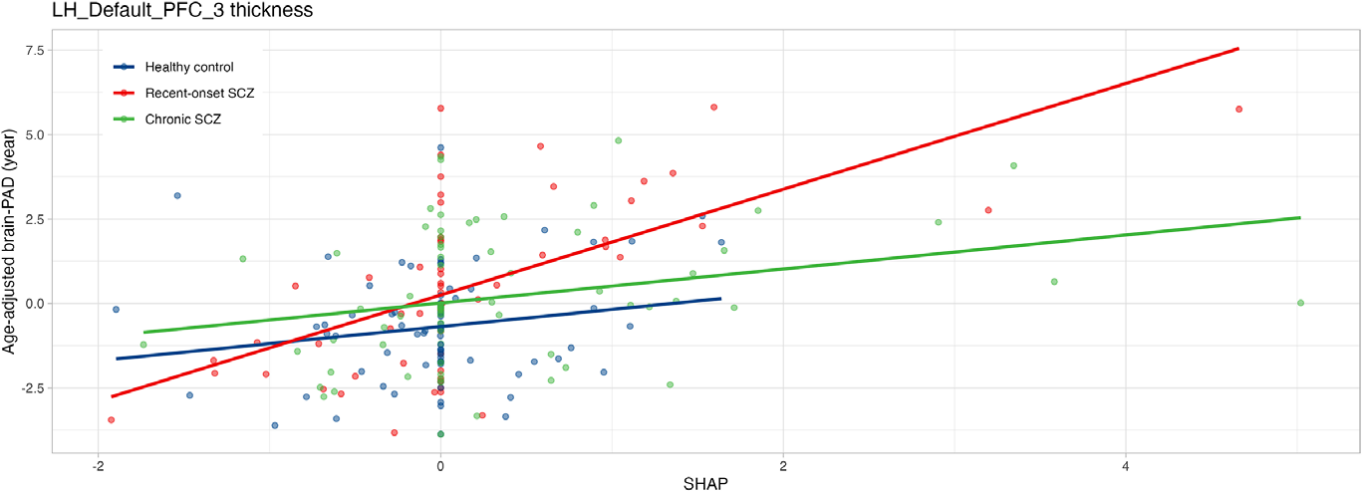
Group-by-SHAP value interactions in thickness of the left prefrontal cortex in the default mode network. Healthy controls, patients with recent-onset schizophrenia, and patients with chronic schizophrenia are represented in blue, red, and green, respectively. Brain-PAD, brain-predicted age difference; PFC, prefrontal cortex; SHAP, SHapley Additive exPlanations; SCZ, schizophrenia.

We selected the top 20 features based on their absolute SHAP values and investigated between-group differences in SHAP values. We also explored group-by-SHAP value interactions for age-adjusted brain-PAD. Supplementary Table 6 shows the top 20 features and the statistics for the group comparisons and interactions, demonstrating a significant between-group difference in the SHAP value of the RH_Cont_PFCl_1_thickness (cortical thickness of the right lateral prefrontal area in the control network) (F = 7.062, FDR p = 0.022) and a significant group-by-SHAP value interaction in the LH_Default_PFC_3_thickness (cortical thickness of the left prefrontal area in the default mode network) (F = 6.198, FDR p = 0.049) (Figure 3). Healthy controls had increased SHAP values of RH_Cont_PFCl_1_thickness compared with patients with chronic schizophrenia (p = 0.044) and recent-onset schizophrenia (p = 0.001) (Supplementary Figure 5).

## Discussion

In this study, using structural MRI data from over 10,000 healthy individuals, we developed brain age prediction models. These models were validated on an independent test dataset comprising healthy controls, patients with recent-onset schizophrenia, and patients with chronic schizophrenia. The results indicated that patients with recent-onset schizophrenia and those with chronic schizophrenia exhibited increased brain-PAD of 1.2 and 0.9 years, respectively, compared to healthy controls. Regarding associations with clinical variables, a negative correlation between brain-PAD and FSIQ was observed in patients with chronic schizophrenia; however, this association did not remain significant after correction for multiple comparisons. Feature importance, as assessed based on SHAP value, was compared across the groups, and group-by-SHAP value interactions for age-adjusted brain-PAD were analyzed. A significant between-group difference in SHAP value was identified for the thickness of the right lateral prefrontal area in the control network, and a significant interaction effect was observed for the thickness of the left prefrontal area in the default mode network.

Our findings revealed that patients with recent-onset schizophrenia and chronic schizophrenia exhibited increased brain-PAD of 1.2 and 0.9 years, respectively, compared to healthy controls. The differences in brain-PAD between the patient and control groups in our study were smaller than those reported in previous studies (Hajek et al., 2019; Nenadic, Dietzek, Langbein, Sauer, & Gaser, 2017; Shahab et al., 2019), where patients with schizophrenia exhibited increased brain-PADs ranging from 2.6 to 7.8 years compared to healthy controls. In a large-scale study, Constantinides et al. also reported a higher brain-PAD of 3.55 years in patients with schizophrenia than in healthy controls (Constantinides et al., 2023). The discrepancies may be attributed to several factors, including differences in the clinical characteristics of study populations, neuroimaging features used for model development, and variations in brain age prediction models (Han, Kim, Lee, & Lee, 2022). The greater increase in brain-PAD in recent-onset schizophrenia than in chronic schizophrenia observed in the present study aligns with previous findings by Schnack et al. (H. G. Schnack et al., 2016), who reported that the acceleration rate of brain-PAD in schizophrenia slows to a normal rate approximately 5 years after illness onset. However, our findings are in contrast to those of Kim et al. (Kim et al., 2023; Kim et al., 2024), who reported a greater increase in brain-PAD in patients with treatment-resistant schizophrenia compared to those with first-episode schizophrenia. This difference may be attributed to structural brain abnormalities in treatment-resistant schizophrenia being compounded by the effects of disease progression (e.g. illness duration) and distinct biological characteristics specific to treatment-resistant schizophrenia (Potkin et al., 2020). Our finding of a more pronounced increase in brain-PAD during the early phase of the illness than the later phase supports the neurodevelopmental hypothesis of schizophrenia pathophysiology (Fatemi & Folsom, 2009). Although brain age as a single metric offers a simplified approach to understanding the complex structural abnormalities of the brain, the underlying causes of accelerated brain aging remain unclear, warranting further investigation.

We observed a potential association between brain-PAD and general intelligence in patients with chronic schizophrenia; however, this association did not remain statistically significant after adjusting for multiple comparisons. The lack of significance may be attributed to the small sample size of the present study. Moreover, as Kaufmann et al. highlighted, a limitation of brain-PAD is its reliance on a summary metric, which overlooks regional contributions of structural brain abnormalities (Kaufmann et al., 2019). Therefore, it is necessary to consider the spatial and regional characteristics of brain abnormalities to better elucidate the clinical relevance of brain-PAD. Further research is required to clarify the associations between brain-PAD, cognitive deficits, and negative symptoms in schizophrenia, leveraging larger sample sizes and more targeted study designs.

We calculated SHAP values to investigate the extent to which individual features impact brain-PAD across the three groups. Significant between-group differences in SHAP values were observed in the right lateral prefrontal area within the control network, and group-by-SHAP value interactions were found in the left prefrontal area within the default mode network. Regarding the significant group-by-SHAP value interaction effects, our findings indicated group differences in the associations between the contribution of cortical thickness in the left prefrontal area to the model and brain-PAD. These findings should be interpreted with caution, as they do not suggest a direct link between structural changes in the left prefrontal area and accelerated brain aging in schizophrenia. This may be due to the limited understanding of how changes in this region influence the model. The right prefrontal area is widely recognized for its role in cognitive control and executive function, as part of the frontoparietal network (Friedman & Robbins, 2022). Hypoactivation of the right prefrontal area is associated with negative symptoms (Fuentes-Claramonte et al., 2022), and reduced connectivity between the right prefrontal area and the inferior parietal area within the frontoparietal network has also been observed in schizophrenia (Ćurčić-Blake, Kos, & Aleman, 2022). The left prefrontal area is involved in the default mode network which is associated with various brain functions, including self-referential processing, social cognition, and episodic and autobiographical memory (Menon, 2023). Extensive evidence highlights abnormalities in the default mode network in schizophrenia (Hu et al., 2017), with the left prefrontal area specifically implicated in thought disorder (Marumo et al., 2014) and impaired memory encoding and retrieval (Ragland et al., 2009). Ballester et al. identified the total gray matter volume as the most predictive feature for brain age in schizophrenia, reporting that the total gray matter volume did not show significant interactions with brain-PAD in a non-psychotic depression dataset (Ballester et al., 2023). Our findings differed from those of Ballester et al., as cortical thickness, rather than gray matter volume, in the left or right prefrontal area was associated with between-group differences and group-by-SHAP value interactions in our study. In addition to the differences in neuroimaging features and prediction models, it is important to note that our study categorized patients into the recent-onset schizophrenia and chronic schizophrenia groups, which may further explain these discrepancies. Our results suggest that the regional impact of structural abnormalities on brain age prediction may vary with illness duration. However, the cross-sectional design of this study limits the interpretation of these findings. Longitudinal studies with larger sample sizes are needed to further explore these relationships and address this issue comprehensively.

Our findings highlight the critical role of sophisticated machine learning approaches in accurately predicting brain age based on structural MRI data, revealing valuable insights into the computational techniques that can effectively capture the complex morphological signatures of brain aging. The remarkable performance of ensemble methods, particularly the CatBoost_r9_BAG_L2 model with an MAE of 6.718 years, substantiates the growing recognition of ensemble learning’s potential in neuroimaging analysis. These results demonstrate that ensemble techniques can effectively mitigate individual model limitations by integrating diverse algorithmic perspectives. Similarly, the LightGBM-based models, specifically LightGBMLarge_BAG_L2 and LightGBMXT_BAG_L2, demonstrated comparable performance with MAEs of 6.674 and 6.731 years, respectively. Conversely, simpler algorithms, such as KNeighborsDist_BAG_L1 and KNeighborsUnif_BAG_L1, exhibited a significantly lower performance, with MAEs of 12.783 and 12.810 years, respectively. Simple distance-based algorithms struggle to navigate high-dimensional, non-linear morphological variations across different brain structures. This limitation underscores the necessity of sophisticated machine-learning approaches that can capture subtle, complex interactions between brain structural features. The WeightedEnsemble models, particularly the L3 architecture, demonstrated the potential of hierarchical model stacking. By strategically combining multiple base models, these approaches can comprehensively represent the multifaceted nature of brain aging. The L3 model’s superior performance suggests that deeper ensemble architectures can more effectively integrate diverse feature representations and predictive signals. Our results align with and build upon the findings of Zhang et al. and Li et al. (Li et al., 2024; Z. Zhang et al., 2022), reinforcing the consensus that ensemble methods offer significant advantages in neuroimaging-based predictive modeling. The consistent outperformance of gradient boosting and ensemble approaches across different studies indicates a robust methodological trend in brain age prediction research.

We utilized a large-scale dataset of structural MRI findings of healthy individuals to develop brain age prediction models, which significantly enhanced their performance and accuracy. The clinical implications of brain-PAD were investigated considering illness duration in the patient group, providing insights into the clinical characteristics of schizophrenia in the context of brain-PAD interpretation. However, certain limitations of the present study must be acknowledged. First, the independent test dataset was relatively small and consisted of three different cohorts, with participants recruited based on MRI data obtained using different scanners and imaging parameters. Although we applied a harmonization method to standardize individual datasets before inputting them into the prediction model, this variability should be considered when interpreting our findings. Second, the chronic schizophrenia group included patients with treatment-responsive and those with treatment-resistant schizophrenia. Previous studies have reported distinct biological characteristics of the brains of patients with treatment-resistant schizophrenia (Potkin et al., 2020). This heterogeneity likely contributed to the relatively smaller increase in brain-PAD observed in the chronic schizophrenia group, reflecting a mixture of two biologically distinct subgroups. Third, the number of clinical variables was limited due to the integration of three separate cohorts into a single test dataset. A more comprehensive assessment of psychiatric symptoms and neurocognitive functions is necessary to deepen the understanding of the clinical implications of the changes in brain-PAD in patients with schizophrenia. Fourth, educational attainment has been recognized as a proxy for cognitive reserve and is associated with brain-PAD. Steffener et al. (Steffener et al., 2016) reported that a higher educational level was associated with a lower brain-PAD, suggesting a protective effect on accelerated brain aging. In the present study, the test dataset comprised three cohorts evaluated using different tools, which limited the inclusion of educational attainment as a covariate in the analyses. Further studies with more rigorous adjustments for confounding factors are needed to reveal more accurate measures of brain aging in schizophrenia. Fifth, we used the Schaefer atlas for cortical parcellation which offers neurobiologically meaningful features by capturing intrinsic functional connectivity patterns (Schaefer et al., 2018). However, its application to structural MRI data warrants careful consideration. Functional atlases are primarily designed to reflect connectivity patterns, which may not always correspond directly to the brain’s structural organization. Despite these concerns, we selected the Schaefer atlas based on its established utility in previous studies (Hansen et al., 2024; Kuchenhoff et al., 2024; Luppi et al., 2024; Serio et al., 2024; Valk et al., 2020; L. Zhang et al., 2025), where it demonstrated robust performance in extracting biologically relevant features even from structural data. Finally, the cross-sectional design of this study limits the ability to infer causal relationships.

In this study, we utilized a large-scale structural MRI dataset of healthy individuals to develop accurate brain age prediction models and showed increased brain-PADs in patients with schizophrenia, particularly during the early phase of illness. These findings support the neurodevelopmental hypothesis and highlight accelerated brain aging as a potential biomarker for schizophrenia. While brain-PAD showed potential associations with clinical characteristics such as general intelligence, these associations did not remain significant after correcting for multiple comparisons, possibly due to the small sample size and heterogeneity within the chronic schizophrenia group. Feature importance analyzed based on SHAP values revealed regional variations in the contributions of cortical thickness in brain age prediction across the groups, emphasizing the need to consider spatial and regional characteristics when interpreting brain-PADs. Future longitudinal studies with larger, clinically homogeneous samples and comprehensive symptom and cognitive function assessments are necessary to validate these findings and refine the clinical utility of brain-PAD in understanding the pathophysiology of schizophrenia.

## Supporting information

Joo et al. supplementary material Joo et al. supplementary material

## Supplementary material

The supplementary material for this article can be found at http://doi.org/10.1017/S0033291725000285.
