# Supplementary material for: Disparities in accelerated brain aging in recent-onset and chronic schizophrenia
Source: Psychol Med. 2025 Feb 24;55:e60. doi: 10.1017/S0033291725000285 (PMC12080660; doi:10.1017/S0033291725000285)
Supplement: Joo et al. supplementary material [file S0033291725000285sup001.docx]

**Supplementary Material**

**Study population – test sample**

We included all participants from Asan Medical Center, a university-affiliated hospital. Each cohort was designed to investigate the neurobiological alterations in patients with schizophrenia or bipolar disorders. Chronologically, we began with the inclusion of participants in the AMC 1 cohort, followed by that in the AMC 2 cohort, and then in the AMC 3 cohort. In each cohort, psychiatric symptoms and cognitive functions were assessed using different tools. Magnetic resonance imaging (MRI) parameters varied across the cohorts, as detailed in the Supplementary Materials. We have provided the specifics of each cohort, including inclusion and exclusion criteria, as well as clinical information in the following sections.

**AMC 1**

An experienced psychiatrist diagnoses patients according to the Diagnostic and Statistical Manual of Mental Disorders IV (DSM-IV) criteria. For all patients, the onset of the first psychotic symptoms, including delusion or hallucination, occurred in ≤ 5 years prior to enrollment. In the control group, subjects and their first-degree relatives had no Axis I psychiatric diagnoses based on the DSM-IV-TR criteria. All subject were right-handed and aged between 20 and 40 years. Exclusion criteria included diseases that affect the functioning of the brain or the inability to undergo complete neuropsychological testing or MRI scanning. We excluded 20 subjects owing to poor image quality, incidental brain lesions, or a change in the original diagnosis to other psychotic disorders (e.g., bipolar disorder) upon re-evaluation 1–6 months after enrollment. All subjects provided written informed consent before enrollment, and ethical approval for the study was obtained from the Institutional Review Board of Asan Medical Center (IRB File No. 2012-0485).

Assessment of symptoms, neurocognition, and social cognition was completed within a week from the date of the MRI. All subjects were evaluated based on the age- and sex-adjusted short form of the Wechsler Adult Intelligence Scale-Third edition (WAIS-III) and the video-based social cognition scale (VISC). The age- and sex-adjusted short form of the WAIS consists of six subtests, including digit span, vocabulary, arithmetic, picture arrangement, block design, and digit symbol. The VISC is a scale for evaluating social cognition based on 20 video clips portraying frequently experienced social interactions in real life. Patients were asked to point out socially unnatural behavior or speech or to state the reasons behind lies portrayed by actors or actresses. Each question is scored on a 0–2 scale, with a maximum possible score of 40. Patients' psychiatric symptoms were rated using the Positive and Negative Syndrome Scale (PANSS).

**AMC 2**

Participants were recruited between August 2017 and February 2020. The patient group included those diagnosed with schizophrenia or bipolar disorders. One of the main objectives of forming this cohort was to investigate structural and functional similarities and differences between those with schizophrenia and bipolar disorders. Diagnoses in the patient group were made using the Structured Clinical Interview for DSM Disorders (SCID). The control group comprised participants who met the following criteria: (1) no history of Axis I diagnosis according to the SCID and (2) no history of Axis I diagnosis in first-degree relatives according to the SCID. Additional inclusion criteria for both groups were (1) age between 20 and 55 years, (2) right-handedness, (3) Full-Scale Intelligence Quotient (FSIQ) score above 80, and (4) absence of any physical illness that could interfere with brain function. After visual inspection of the MRI scans, the final sample included 27 patients with schizophrenia, 24 patients with bipolar disorder, and 55 healthy controls. Written informed consent was obtained from all participants prior to enrollment, and the study received ethical approval from the Institutional Review Board of Asan Medical Center (IRB File No. 2017-0839).

Neurocognitive function was assessed using the Cogstate Brief Battery, which evaluates seven cognitive domains, including processing speed, attention, working memory, visual learning and memory, verbal learning and memory, executive functions, and social cognition.

**AMC 3**

We recruited participants from June 2021 to December 2023. The inclusion criteria were 1) a diagnosis of schizophrenia made according to the Diagnostic and Statistical Manual of Mental Disorders V (DSM-V), 2) an illness duration of > 5 years, and 3) right-handedness. Participants were excluded based on the following criteria: 1) a history of intellectual disability; 2) a history of substance abuse or dependency in the last 6 months; 3) a history of head trauma leading to > 3 min of unconsciousness; 4) neurological disorders; and 5) unstable diseases that may interfere with brain functioning. All participants provided written informed consent before enrollment. Among 69 patients with schizophrenia who were initially enrolled, 14 withdrew their consent, resulting in 55 patients completing the study protocol. This study received approval from the IRB of the AMC (IRB No. 2021-1128, 2022-1193) and was conducted according to the Declaration of Helsinki. The final study population consisted of 52 patients with schizophrenia, excluding 3 owing to poor image quality (n=2) and major neurological malformation (n=1).

Interviews and evaluations of the participants were conducted 1 week before the MRI, and clinical information, including previous and current illness history, family history, and antipsychotic treatment history, was obtained. The severity of psychiatric symptoms was assessed using the PANSS, and cognitive functions were evaluated using a shorter version of the Wechsler Adult Intelligence Scale-Fourth edition (WAIS-IV), Rey-Kim Memory Test, and Kims Frontal-Executive Neuropsychological Test.

**ComBat harmonization**

To assess scanner variability, we carefully constructed balanced groups of 510 samples each from 1.5T and 3T scanners. We first confirmed that the age distributions between these groups were comparable using Wilcoxon signed-rank test (z = 60069.5, p = 0.737). The non-parametric Wilcoxon signed-rank test was chosen based on the Shapiro-Wilk test results, which indicated that the data did not follow a normal distribution. Our analysis of ComBat's effectiveness in reducing scanner-related variability revealed several key findings. Before harmonization, we identified significant differences between 1.5T and 3T scans across multiple ROIs (Supplementary Table 4). Specifically, 119 features showed statistically significant differences between scanner types. After applying ComBat harmonization, 84 of these features no longer showed significant differences, demonstrating substantial improvement. However, 50 features remained statistically different, which may reflect either limitations of the harmonization method or genuine individual variability in the sample population. To illustrate these effects visually, we have included distribution plots (Supplementary Figures 1-3) showing cortical thickness, surface area, and subcortical volume for three representative ROIs before and after harmonization. These visualizations demonstrate the convergence of distributions after ComBat application, providing clear evidence of reduced scanner-related variability.

**Model development – Algorithms**

KNeighborsUnif (Cover and Hart 1967): A classic k-nearest neighbors (k-NN) algorithm, which predicts the output of a query point based on the majority class or average value of its k nearest neighbors in the feature space. In KNeighborsUnif, all neighbors contribute equally to the prediction, regardless of their distance from the query point.

KNeighborsDist (Dudani 1976): A distance-weighted variant of k-NN, which assigns higher weights to closer neighbors, enhancing the relevance of nearby points in prediction.

ExtraTrees (XT) (Geurts, Ernst et al. 2006): An ensemble method that constructs multiple decision trees by introducing randomness in node splitting; each split point is selected randomly for each feature, enhancing generalization and reducing variance.

RandomForest (Breiman 2001): A widely-used ensemble technique that builds multiple decision trees. By selecting random subsets of features and data points for each tree it mitigates overfitting and variance. The final prediction aggregates the outputs from all trees.

XGBoost (Chen and Guestrin 2016): A scalable, efficient implementation of gradient boosting that builds trees sequentially, with each tree correcting errors from previous ones. The final prediction sums contributions from all trees.

LightGBM (Ke, Meng et al. 2017): A gradient boosting framework known for efficiency and scalability. Innovations such as histogram-based learning, leaf-wise growth, and exclusive feature bundling improve training speed and memory efficiency.

LightGBMXT (Ke, Meng et al. 2017): A LightGBM variant incorporating the XT method, introducing additional randomness by selecting split points randomly rather than based on information gain, which further reduces overfitting.

LightGBMLarge (Ke, Meng et al. 2017): A LightGBM version optimized for large datasets with extensive features. It incorporates additional regularization and optimization techniques to maintain performance and prevent overfitting on vast datasets.

Categorical boosting (CatBoost) (Prokhorenkova, Gusev et al. 2018): A gradient boosting algorithm designed to handle categorical features effectively without requiring extensive preprocessing like one-hot encoding. CatBoost’s ordered boosting and permutation-based approach reduces prediction shift and overfitting.

NeuralNetFastAI (Howard and Gugger 2020): A deep learning model implemented in the FastAI library, specialized for tabular datasets. It leverages efficient architectures optimized for structured data.

NeuralNetTorch (Erickson, Mueller et al. 2020): A deep learning model implemented using the PyTorch framework, suitable for flexible deep learning experiments with custom neural architectures.

WeightedEnsemble (Erickson, Mueller et al. 2020): An ensemble method that combines predictions from multiple base models, assigning each model a weight based on its performance, enhancing overall accuracy.

**Model development – Bagging and Stacking**

Bootstrap aggregating (Bagging) (Breiman 1996) is an ensemble method designed to reduce model variance by training multiple base models on different bootstrap samples, which are randomly generated subsets of the original dataset. The final prediction is obtained by aggregating the outputs of all base models, typically through majority voting for classification or averaging for regression tasks. Bagging is particularly effective for high-variance models, enhancing stability and generalization.

Stacking (Wolpert 1992) is an ensemble technique that combines the predictions of multiple base models using a meta-model. In this approach, base models (Level 0) are first trained on the original dataset, and their predictions are used as input features for a higher-level meta-model (Level 1). The meta-model synthesizes these predictions to produce the final output, leveraging the strengths of diverse base models to capture complex data patterns. To prevent overfitting, k-fold cross-validation is commonly used during training. Stacking is especially beneficial for tasks where a combination of algorithms can capture complementary patterns in the data.

**Supplementary Table 1.** Demographics and clinical characteristics of the training sample

| Dataset | Number of participants | Age, mean (SD), year | Male sex, n (%) | Age range, year |
| --- | --- | --- | --- | --- |
| DLBS (Park, Carp et al. 2012) | 314 | 54.00 (20.04) | 117 (37.26) | 20–89 |
| fcon1000 (Biswal, Mennes et al. 2010) | 987 | 30.10 (14.40) | 554 (53.01) | 7–85 |
| HBN (Alexander, Escalera et al. 2017) | 971 | 10.33 (3.37) | 628 (64.67) | 5.05–21.22 |
| HCP (Van Essen, Smith et al. 2013) | 1,061 | 28.75 (3.67) | 486 (45.80) | 22–37 |
| IXI (https://brain-development.org/) | 562 | 48.65 (16.47) | 250 (44.48) | 19.98–86.32 |
| MCIC (Gollub, Shoemaker et al. 2013) | 94 | 32.63 (11.97) | 64 (68.08) | 18–60 |
| AOMIC (Snoek, van der Miesen et al. 2021) | 209 | 22.18 (1.79) | 89 (42.58) | 18.25–26.25 |
| BGSP (Holmes, Hollinshead et al. 2015) | 1,493 | 21.53 (2.89) | 632 (42.33) | 19–35 |
| BNU (Tian, Wang et al. 2011) | 180 | 21.22 (1.93) | 73 (40.55) | 17–28 |
| Cam-CAN (Shafto, Tyler et al. 2014) | 631 | 54.93 (18.38) | 312 (49.52) | 18–88 |
| COBRE (Aine, Bockholt et al. 2017) | 93 | 37.63 (11.66) | 67 (72.04) | 18–65 |
| CoRR (Zuo, Anderson et al. 2014) | 1,373 | 24.60 (13.66) | 692 (50.40) | 6–84 |
| DecNef (Tanaka, Yamashita et al. 2021) | 949 | 36.59 (15.52) | 538 (56.69) | 18–80 |
| NARPS (Botvinik-Nezer, Iwanir et al. 2019) | 108 | 25.54 (3.59) | 48 (44.44) | 18–37 |
| NPC (Sunavsky and Poppenk 2020) | 65 | 26.55 (4.30) | 29 (42.85) | 20–35 |
| NUSDAST (Kogan, Alpert et al. 2016) | 98 | 31.95 (13.76) | 53 (37.85) | 14–67 |
| OASIS-3 (LaMontagne, Benzinger et al. 2019) | 714 | 68.68 (8.91) | 306 (42.85) | 42–95 |
| SALD (Wei, Zhuang et al. 2018) | 494 | 45.18 (17.44) | 187 (37.85) | 19–80 |
| SLIM (Liu, Wei et al. 2017) | 387 | 20.07 (1.26) | 244 (63.04) | 17–27 |
| UCLA CNP (Poldrack, Congdon et al. 2016) | 125 | 31.52 (8.79) | 66 (52.80) | 21–50 |
| Total | 10,938 | 32.61 (19.14) | 5,246 (47.96) | 5.05–95 |

**Supplementary Table 2.** Scanners and T1-weighted MRI parameters for the training samples

| Dataset | Scanners | Protocol parameters |
| --- | --- | --- |
| DLBS | Philips Achieva 3T | TR = 8.27 ms, TE = 3.82 ms, voxel size = 1 × 1 × 1 mm |
| HBN | 1.5 T Siemens Avanto  3T Siemens Tim Trio | 1.5T: TR = 2730 ms, TE = 1.64 ms, TI = 1000 ms, flip angle = 7°, voxel size = 1.0 × 1.0 × 1.0 mm, 176 slices  3T: TR = 2500 ms, TE = 3.15 ms, TI = 1060 ms, flip angle = 8°, voxel size = 0.8 × 0.8 × 0.8 mm, 224 slices |
| IXI | Philips 3T (Hammersmith Hospital), Philips 1.5T (Guy's Hospital), GE 1.5T (Institute of Psychiatry) | TR = 9.6 ms, TE = 4.6 ms, flip angle = 8.0°, slices = 208 |
| HCP | Customized Siemens 3T "Connectome Skyra" | TR = 2400 ms, TE = 2.14 ms, TI = 1000 ms, flip angle = 8°, field of view (FOV) = 224 × 224 mm, voxel size = 0.7 mm isotropic, 256 sagittal slices |
| Fcon1000 | Various 3T (n = 970), Various 1.5T (n = 123) | TR = 1.5–3 s, voxel size = 2–4 mm, scan duration = 2.2–20 min |
| MCIC | 1.5T and 3T Siemens | TR = 2530 ms (3T), 12 ms (1.5T), TE = 3.79 ms (3T), 4.76 ms (1.5T), flip angle = 7° (3T), 20° (1.5T), voxel size = 0.625 × 0.625 × 1.5 mm |
| AOMIC | Philips 3T | TR = 2250 ms, TE = 2.99 ms, flip angle = 9°, voxel size = 1 mm isotropic |
| BGSP | 3T Siemens Tim Trio | TR = 2.2 s, TE = 1.5/3.4/5.2/7.0 ms, flip angle = 7°, TI = 1.1 s, slice thickness = 1.2 mm, resolution = 1.2 × 1.2 × 1.2 mm, slices = 144, duration = 2'12" |
| BNU | 3T Siemens | TR= 2000 ms; TE=30 ms; slices=33; thickness= 3 mm; gap, 0.6 mm; FOV= 200 × 200 mm^2^; resolution, 64 × 64; flip angle=90° |
| Cam-CAN | 3T Siemens TIM Trio | TR = 2250 ms, TE = 2.99 ms, TI = 900 ms, flip angle = 9°, voxel size = 1 mm isotropic |
| COBRE | 3T Siemens TIM Trio | TR = 2.53 s, TE = 1.64, 3.5, 5.36, 7.22, 9.08 ms, TI = 1.2 s, flip angle = 7°, voxel size = 1 mm isotropic, number of excitations = 1, slice thickness = 1 mm, FOV = 256 mm, resolution = 256 × 256 |
| CoRR | Various 3T scanners | TR = 7.788–2530 ms, TE = 3.39 ms, TI = 450–1100 ms, flip angle = 7–12°, FOV = 256 × 256 mm, voxel size = 1.0 × 1.0 × 1.0 mm, 128–256 sagittal slices, Headcoil 1-32 channel |
| DecNef | Various 3T scanners | <https://www.nature.com/articles/s41597-021-01004-8> |
| NARPS | 3T Siemens Prisma | TR = 1000 ms, TE = 30 ms, flip angle = 68°, FOV =  212 × 212 mm, slice thickness = 2 mm, 64 slices and gap = 0.4 mm |
| NPC | Magnetom Tim Trio; Siemens Healthcare | resolution = 0.7 × 0.7 × 0.7 mm, matrix = 320 × 320, slices = 256 AC-PC transverse, TR = 2400 ms, TE = 2.13 ms, flip angle = 8°, echo spacing = 6.5 ms |
| NUSDAST | 1.5T Vision scanner platform (Siemens Medical Systems) | TR = 20 ms, TE = 5.4 ms, flip angle = 30°, ACQ = 1, 256 × 256 matrix, 1 × l mm in-plane resolution, 180 slices, slice thickness 1 mm, 13:30 min scan time |
| OASIS-3 | Siemens TimTrio 3.0T, Siemens Sonata 1.5T | TE = 3.1 ms, TR = 3 s, TI = 0.8 s, acquisition resolution = × 1 × 1.2 mm |
| SALD | 3.0-T Siemens Trio MRI scanner | T1-weighted anatomical images (repetition time = 1,900 ms, echo time = 2.52 ms, inversion time = 900 ms, flip angle = 90°, resolution matrix = 256×256, slices = 176, thickness = 1.0 mm, and voxel size = 1 × 1 × 1 mm^3^ |
| SLIM | 3.0-T Siemens Trio MRI scanner | repetition time = 1,900 ms, echo time = 2.52 ms, inversion time = 900 ms, flip angle = 9°, resolution matrix = 256 × 256, slices = 176, thickness = 1.0 mm, voxel size = 1 × 1 × 1 mm^3^ |
| UCLA CNP | 3T Siemens Trio scanners | TR = 1.9 s, TE = 2.26 ms, FOV = 250 mm, matrix = 256 × 256, sagittal plane, slice thickness = 1 mm, 176 slices |

FOV, field of view; MRI, magnetic resonance imaging

**Supplementary Table 3.** Scanners and T1-weighted MRI parameters for the test samples

| Dataset | Scanners | Protocol parameters |
| --- | --- | --- |
| AMC 1 | 3T Philips Achieva | T1-weighted structural images were acquired with a 8-channel SENSE head-coil (TE = 4.6 ms, TR = 9.0 ms, voxel size = 1.0 × 1.0 × 1.0 mm, FOV = 240 × 240 × 170 mm, flip angle = 8°) |
| AMC 2 | 3T Philips Ingenia | TE = 4.6 ms, TR = 9.9 ms, voxel size = 1.0 × 1.0 × 1.0 mm, FOV = 240 × 240 × 170 mm, flip angle = 8° |
| AMC 3 | 3T Philips Ingenia CX | Sagittal T1-weighted structural images were acquired with a 32-channel dStream head coil and 3D, FFE sequence (TE = 2.9 ms, TR = 6.5 ms, flip angle = 9°, FOV (RL, AP, FH) = 211 × 256 × 256 mm, voxel size = 1.0 × 1.0 × 1.0 mm, 211 slices, slice thickness = 1 mm). |

FOV, field of view; MRI, magnetic resonance imaging

**Supplementary Table 4.** Wilcoxon signed-rank test results comparing 1.5T and 3T samples before and after harmonization

| Feature | Before Harmonization | | After Harmonization | |
| --- | --- | --- | --- | --- |
|  | Statistic | p-value | Statistic | p-value |
| LH_Vis_1_thickness | 58198 | 0.043 | 64321 | 0.802 |
| LH_Vis_2_thickness | 35134 | <0.001 | 54177 | <0.001 |
| LH_Vis_3_thickness | 45481 | <0.001 | 49869 | <0.001 |
| LH_Vis_4_thickness | 58340 | 0.040 | 58833 | 0.057 |
| LH_Vis_5_thickness | 44364 | <0.001 | 56469 | 0.009 |
| LH_Vis_6_thickness | 50497 | <0.001 | 56586 | 0.010 |
| LH_Vis_7_thickness | 44892 | <0.001 | 60469 | 0.159 |
| LH_Vis_8_thickness | 49773 | <0.001 | 59517 | 0.090 |
| LH_Vis_9_thickness | 44324 | <0.001 | 57558 | 0.022 |
| LH_SomMot_1_thickness | 47425 | <0.001 | 58399 | 0.042 |
| LH_SomMot_2_thickness | 60005 | 0.122 | 62759 | 0.472 |
| LH_SomMot_3_thickness | 57937 | 0.042 | 62190 | 0.373 |
| LH_SomMot_4_thickness | 31195 | <0.001 | 62810 | 0.481 |
| LH_SomMot_5_thickness | 24600 | <0.001 | 59948 | 0.118 |
| LH_SomMot_6_thickness | 20323 | <0.001 | 60830 | 0.194 |
| LH_DorsAttn_Post_1_thickness | 47621 | <0.001 | 54625 | 0.001 |
| LH_DorsAttn_Post_2_thickness | 45539 | <0.001 | 62698 | 0.461 |
| LH_DorsAttn_Post_3_thickness | 47523 | <0.001 | 61382 | 0.257 |
| LH_DorsAttn_Post_4_thickness | 44784 | <0.001 | 63597 | 0.640 |
| LH_DorsAttn_Post_5_thickness | 50716 | <0.001 | 61001 | 0.212 |
| LH_DorsAttn_Post_6_thickness | 45291 | <0.001 | 61515 | 0.274 |
| LH_DorsAttn_PrCv_1_thickness | 37901 | <0.001 | 62497 | 0.425 |
| LH_DorsAttn_FEF_1_thickness | 28943 | <0.001 | 63343 | 0.586 |
| LH_SalVentAttn_ParOper_1_thickness | 50417 | <0.001 | 61279 | 0.244 |
| LH_SalVentAttn_FrOperIns_1_thickness | 51812 | <0.001 | 62921 | 0.502 |
| LH_SalVentAttn_FrOperIns_2_thickness | 59918 | 0.133 | 62297 | 0.391 |
| LH_SalVentAttn_PFCl_1_thickness | 63446 | 0.608 | 61790 | 0.312 |
| LH_SalVentAttn_Med_1_thickness | 53772 | <0.001 | 59272 | 0.077 |
| LH_SalVentAttn_Med_2_thickness | 51628 | <0.001 | 55749 | 0.004 |
| LH_SalVentAttn_Med_3_thickness | 47850 | <0.001 | 62631 | 0.448 |
| LH_Limbic_OFC_1_thickness | 56571 | 0.017 | 53819 | <0.001 |
| LH_Limbic_TempPole_1_thickness | 61916 | 0.369 | 55319 | 0.003 |
| LH_Limbic_TempPole_2_thickness | 62964 | 0.511 | 58049 | 0.032 |
| LH_Cont_Par_1_thickness | 49870 | <0.001 | 62950 | 0.508 |
| LH_Cont_PFCl_1_thickness | 58796 | 0.056 | 65009 | 0.965 |
| LH_Cont_pCun_1_thickness | 49523 | <0.001 | 63299 | 0.577 |
| LH_Cont_Cing_1_thickness | 52011 | <0.001 | 58187 | 0.036 |
| LH_Default_Temp_1_thickness | 47163 | <0.001 | 60457 | 0.158 |
| LH_Default_Temp_2_thickness | 48542 | <0.001 | 60107 | 0.129 |
| LH_Default_Par_1_thickness | 48167 | <0.001 | 64318 | 0.802 |
| LH_Default_Par_2_thickness | 50619 | <0.001 | 63544 | 0.629 |
| LH_Default_PFC_1_thickness | 64783 | 0.911 | 65120 | 0.992 |
| LH_Default_PFC_2_thickness | 62854 | 0.538 | 59715 | 0.102 |
| LH_Default_PFC_3_thickness | 64666 | 0.944 | 55618 | 0.004 |
| LH_Default_PFC_4_thickness | 55719 | 0.008 | 51687 | <0.001 |
| LH_Default_PFC_5_thickness | 59142 | 0.083 | 64001 | 0.729 |
| LH_Default_PFC_6_thickness | 45828 | <0.001 | 60689 | 0.180 |
| LH_Default_PFC_7_thickness | 53469 | <0.001 | 63319 | 0.581 |
| LH_Default_pCunPCC_1_thickness | 55475 | <0.001 | 58861 | 0.058 |
| LH_Default_pCunPCC_2_thickness | 50435 | <0.001 | 58608 | 0.049 |
| RH_Vis_1_thickness | 47300 | <0.001 | 60227 | 0.139 |
| RH_Vis_2_thickness | 39738 | <0.001 | 50922 | <0.001 |
| RH_Vis_3_thickness | 47571 | <0.001 | 58495 | 0.045 |
| RH_Vis_4_thickness | 57379 | 0.019 | 53162 | <0.001 |
| RH_Vis_5_thickness | 44180 | <0.001 | 54856 | 0.001 |
| RH_Vis_6_thickness | 52790 | <0.001 | 51752 | <0.001 |
| RH_Vis_7_thickness | 50498 | <0.001 | 58299 | 0.039 |
| RH_Vis_8_thickness | 50742 | <0.001 | 55635 | 0.004 |
| RH_SomMot_1_thickness | 44753 | <0.001 | 61335 | 0.251 |
| RH_SomMot_2_thickness | 56191 | 0.007 | 64228 | 0.781 |
| RH_SomMot_3_thickness | 55233 | 0.002 | 63424 | 0.603 |
| RH_SomMot_4_thickness | 30102 | <0.001 | 60919 | 0.203 |
| RH_SomMot_5_thickness | 30628 | <0.001 | 63342 | 0.586 |
| RH_SomMot_6_thickness | 27080 | <0.001 | 58812 | 0.056 |
| RH_SomMot_7_thickness | 42109 | <0.001 | 62995 | 0.517 |
| RH_SomMot_8_thickness | 18190 | <0.001 | 60364 | 0.150 |
| RH_DorsAttn_Post_1_thickness | 60949 | <0.001 | 61769 | 0.309 |
| RH_DorsAttn_Post_2_thickness | 40769 | <0.001 | 64469 | 0.837 |
| RH_DorsAttn_Post_3_thickness | 46166 | <0.001 | 64293 | 0.796 |
| RH_DorsAttn_Post_4_thickness | 47721 | <0.001 | 62559 | 0.436 |
| RH_DorsAttn_Post_5_thickness | 49618 | <0.001 | 63738 | 0.670 |
| RH_DorsAttn_PrCv_1_thickness | 40859 | <0.001 | 58972 | 0.063 |
| RH_DorsAttn_FEF_1_thickness | 32711 | <0.001 | 61043 | 0.217 |
| RH_SalVentAttn_TempOccPar_1_thickness | 51418 | <0.001 | 65094 | 0.985 |
| RH_SalVentAttn_TempOccPar_2_thickness | 45774 | <0.001 | 61492 | 0.271 |
| RH_SalVentAttn_FrOperIns_1_thickness | 56478 | <0.001 | 63851 | 0.695 |
| RH_SalVentAttn_Med_1_thickness | 48772 | <0.001 | 57062 | 0.015 |
| RH_SalVentAttn_Med_2_thickness | 48621 | <0.001 | 65016 | 0.967 |
| RH_Limbic_OFC_1_thickness | 57477 | 0.021 | 51039 | <0.001 |
| RH_Limbic_TempPole_1_thickness | 63389 | 0.596 | 54679 | 0.001 |
| RH_Cont_Par_1_thickness | 51213 | <0.001 | 60066 | 0.126 |
| RH_Cont_Par_2_thickness | 61609 | 0.321 | 64873 | 0.933 |
| RH_Cont_PFCl_1_thickness | 48942 | <0.001 | 53225 | <0.001 |
| RH_Cont_PFCl_2_thickness | 64325 | 0.803 | 63889 | 0.704 |
| RH_Cont_PFCl_3_thickness | 64635 | 0.998 | 63357 | 0.589 |
| RH_Cont_PFCl_4_thickness | 46740 | <0.001 | 60390 | 0.152 |
| RH_Cont_Cing_1_thickness | 56605 | 0.010 | 60354 | 0.149 |
| RH_Cont_PFCmp_1_thickness | 59710 | 0.102 | 56921 | 0.013 |
| RH_Cont_pCun_1_thickness | 48523 | <0.001 | 60053 | 0.125 |
| RH_Default_Par_1_thickness | 60776 | 0.188 | 64898 | 0.939 |
| RH_Default_Temp_1_thickness | 57108 | 0.022 | 56915 | 0.013 |
| RH_Default_Temp_2_thickness | 58849 | 0.068 | 62970 | 0.512 |
| RH_Default_Temp_3_thickness | 48143 | <0.001 | 62093 | 0.358 |
| RH_Default_PFCv_1_thickness | 58819 | 0.067 | 57339 | 0.018 |
| RH_Default_PFCv_2_thickness | 58709 | 0.062 | 63160 | 0.549 |
| RH_Default_PFCdPFCm_1_thickness | 50292 | <0.001 | 61402 | 0.260 |
| RH_Default_PFCdPFCm_2_thickness | 63789 | 0.796 | 64202 | 0.775 |
| RH_Default_PFCdPFCm_3_thickness | 57997 | 0.037 | 62074 | 0.355 |
| RH_Default_pCunPCC_1_thickness | 54172 | 0.001 | 61628 | 0.289 |
| RH_Default_pCunPCC_2_thickness | 45129 | <0.001 | 60626 | 0.174 |
| LH_Vis_1_surface | 63828 | 0.805 | 64007 | 0.730 |
| LH_Vis_2_surface | 57526 | 0.022 | 60257 | 0.141 |
| LH_Vis_3_surface | 64192 | 0.772 | 62227 | 0.379 |
| LH_Vis_4_surface | 58677 | 0.051 | 57148 | 0.016 |
| LH_Vis_5_surface | 63891 | 0.704 | 61687 | 0.297 |
| LH_Vis_6_surface | 63056 | 0.529 | 61495 | 0.272 |
| LH_Vis_7_surface | 64294 | 0.855 | 59470 | 0.087 |
| LH_Vis_8_surface | 58457 | 0.061 | 60900 | 0.201 |
| LH_Vis_9_surface | 65122 | 0.992 | 62811 | 0.481 |
| LH_SomMot_1_surface | 62656 | 0.499 | 63443 | 0.607 |
| LH_SomMot_2_surface | 59260 | 0.089 | 63623 | 0.645 |
| LH_SomMot_3_surface | 59825 | 0.166 | 59656 | 0.098 |
| LH_SomMot_4_surface | 59563 | 0.108 | 57560 | 0.022 |
| LH_SomMot_5_surface | 58170 | 0.035 | 62894 | 0.497 |
| LH_SomMot_6_surface | 55937 | 0.005 | 60795 | 0.190 |
| LH_DorsAttn_Post_1_surface | 59992 | 0.121 | 58441 | 0.043 |
| LH_DorsAttn_Post_2_surface | 63614 | 0.699 | 59775 | 0.106 |
| LH_DorsAttn_Post_3_surface | 58620 | 0.049 | 64369 | 0.813 |
| LH_DorsAttn_Post_4_surface | 61575 | 0.282 | 61972 | 0.339 |
| LH_DorsAttn_Post_5_surface | 62874 | 0.593 | 61880 | 0.325 |
| LH_DorsAttn_Post_6_surface | 60456 | 0.180 | 64514 | 0.847 |
| LH_DorsAttn_PrCv_1_surface | 63068 | 0.531 | 60115 | 0.130 |
| LH_DorsAttn_FEF_1_surface | 59384 | 0.096 | 61417 | 0.261 |
| LH_SalVentAttn_ParOper_1_surface | 60865 | 0.253 | 58318 | 0.040 |
| LH_SalVentAttn_FrOperIns_1_surface | 63565 | 0.633 | 61905 | 0.329 |
| LH_SalVentAttn_FrOperIns_2_surface | 60787 | 0.244 | 63674 | 0.657 |
| LH_SalVentAttn_PFCl_1_surface | 62537 | 0.477 | 62048 | 0.351 |
| LH_SalVentAttn_Med_1_surface | 59898 | 0.114 | 62467 | 0.419 |
| LH_SalVentAttn_Med_2_surface | 62985 | 0.670 | 62181 | 0.372 |
| LH_SalVentAttn_Med_3_surface | 58172 | 0.036 | 58726 | 0.053 |
| LH_Limbic_OFC_1_surface | 64802 | 0.977 | 62812 | 0.482 |
| LH_Limbic_TempPole_1_surface | 64199 | 0.774 | 62092 | 0.358 |
| LH_Limbic_TempPole_2_surface | 64168 | 0.826 | 61572 | 0.282 |
| LH_Cont_Par_1_surface | 63141 | 0.545 | 63053 | 0.528 |
| LH_Cont_PFCl_1_surface | 62146 | 0.407 | 60085 | 0.128 |
| LH_Cont_pCun_1_surface | 63392 | 0.597 | 62018 | 0.346 |
| LH_Cont_Cing_1_surface | 49106 | <0.001 | 61043 | 0.217 |
| LH_Default_Temp_1_surface | 65065 | 0.979 | 62171 | 0.370 |
| LH_Default_Temp_2_surface | 65023 | 0.969 | 63549 | 0.630 |
| LH_Default_Par_1_surface | 62191 | 0.458 | 62442 | 0.415 |
| LH_Default_Par_2_surface | 64797 | 0.975 | 58130 | 0.034 |
| LH_Default_PFC_1_surface | 60432 | 0.230 | 60344 | 0.148 |
| LH_Default_PFC_2_surface | 64620 | 0.873 | 62144 | 0.366 |
| LH_Default_PFC_3_surface | 62489 | 0.423 | 64038 | 0.737 |
| LH_Default_PFC_4_surface | 61447 | 0.265 | 63655 | 0.652 |
| LH_Default_PFC_5_surface | 55460 | 0.004 | 59184 | 0.073 |
| LH_Default_PFC_6_surface | 60028 | 0.123 | 60096 | 0.128 |
| LH_Default_PFC_7_surface | 56236 | 0.007 | 58797 | 0.056 |
| LH_Default_pCunPCC_1_surface | 62539 | 0.575 | 59627 | 0.097 |
| LH_Default_pCunPCC_2_surface | 53936 | <0.001 | 60837 | 0.194 |
| RH_Vis_1_surface | 64732 | 0.899 | 62519 | 0.428 |
| RH_Vis_2_surface | 63134 | 0.544 | 61493 | 0.271 |
| RH_Vis_3_surface | 64604 | 0.929 | 59748 | 0.104 |
| RH_Vis_4_surface | 59112 | 0.081 | 59320 | 0.079 |
| RH_Vis_5_surface | 59894 | 0.151 | 61249 | 0.241 |
| RH_Vis_6_surface | 63348 | 0.587 | 58917 | 0.061 |
| RH_Vis_7_surface | 61739 | 0.305 | 62120 | 0.362 |
| RH_Vis_8_surface | 60425 | 0.202 | 63003 | 0.518 |
| RH_SomMot_1_surface | 62997 | 0.517 | 63617 | 0.644 |
| RH_SomMot_2_surface | 62550 | 0.527 | 64157 | 0.764 |
| RH_SomMot_3_surface | 64461 | 0.895 | 59232 | 0.075 |
| RH_SomMot_4_surface | 54878 | 0.002 | 57658 | 0.024 |
| RH_SomMot_5_surface | 55459 | 0.003 | 58542 | 0.047 |
| RH_SomMot_6_surface | 54846 | 0.002 | 61346 | 0.252 |
| RH_SomMot_7_surface | 59403 | 0.097 | 61431 | 0.263 |
| RH_SomMot_8_surface | 53581 | <0.001 | 62179 | 0.371 |
| RH_DorsAttn_Post_1_surface | 58360 | 0.041 | 59741 | 0.104 |
| RH_DorsAttn_Post_2_surface | 59227 | 0.075 | 60506 | 0.162 |
| RH_DorsAttn_Post_3_surface | 63017 | 0.571 | 63320 | 0.582 |
| RH_DorsAttn_Post_4_surface | 62918 | 0.551 | 64865 | 0.931 |
| RH_DorsAttn_Post_5_surface | 65052 | 0.976 | 62455 | 0.417 |
| RH_DorsAttn_PrCv_1_surface | 64477 | 0.839 | 60713 | 0.182 |
| RH_DorsAttn_FEF_1_surface | 55488 | 0.004 | 60592 | 0.170 |
| RH_SalVentAttn_TempOccPar_1_surface | 64549 | 0.977 | 60042 | 0.124 |
| RH_SalVentAttn_TempOccPar_2_surface | 62497 | 0.469 | 61294 | 0.246 |
| RH_SalVentAttn_FrOperIns_1_surface | 63633 | 0.648 | 60978 | 0.209 |
| RH_SalVentAttn_Med_1_surface | 61278 | 0.275 | 64385 | 0.817 |
| RH_SalVentAttn_Med_2_surface | 54403 | 0.001 | 59596 | 0.095 |
| RH_Limbic_OFC_1_surface | 52880 | <0.001 | 63576 | 0.635 |
| RH_Limbic_TempPole_1_surface | 60680 | 0.179 | 62601 | 0.443 |
| RH_Cont_Par_1_surface | 59557 | 0.092 | 58393 | 0.042 |
| RH_Cont_Par_2_surface | 58583 | 0.048 | 62535 | 0.431 |
| RH_Cont_PFCl_1_surface | 53593 | <0.001 | 62180 | 0.372 |
| RH_Cont_PFCl_2_surface | 64462 | 0.835 | 60576 | 0.169 |
| RH_Cont_PFCl_3_surface | 56565 | 0.009 | 59043 | 0.066 |
| RH_Cont_PFCl_4_surface | 60536 | 0.165 | 60773 | 0.188 |
| RH_Cont_Cing_1_surface | 54921 | 0.002 | 63322 | 0.582 |
| RH_Cont_PFCmp_1_surface | 58275 | 0.038 | 60784 | 0.189 |
| RH_Cont_pCun_1_surface | 60869 | 0.224 | 64444 | 0.831 |
| RH_Default_Par_1_surface | 58376 | 0.041 | 62784 | 0.476 |
| RH_Default_Temp_1_surface | 59751 | 0.121 | 64838 | 0.924 |
| RH_Default_Temp_2_surface | 57895 | 0.041 | 63758 | 0.675 |
| RH_Default_Temp_3_surface | 60887 | 0.256 | 62887 | 0.496 |
| RH_Default_PFCv_1_surface | 61267 | 0.243 | 62820 | 0.483 |
| RH_Default_PFCv_2_surface | 64220 | 0.838 | 57394 | 0.019 |
| RH_Default_PFCdPFCm_1_surface | 59527 | 0.105 | 63358 | 0.589 |
| RH_Default_PFCdPFCm_2_surface | 54741 | 0.001 | 59654 | 0.098 |
| RH_Default_PFCdPFCm_3_surface | 57300 | 0.018 | 63334 | 0.584 |
| RH_Default_pCunPCC_1_surface | 59787 | 0.107 | 58133 | 0.035 |
| RH_Default_pCunPCC_2_surface | 59496 | 0.089 | 64300 | 0.797 |
| Left-Thalamus | 61867 | 0.323 | 54693 | 0.001 |
| Right-Thalamus | 47326 | <0.001 | 59768 | 0.105 |
| Left-Caudate | 45807 | <0.001 | 59910 | 0.115 |
| Right-Caudate | 46353 | <0.001 | 57527 | 0.022 |
| Left-Putamen | 37564 | <0.001 | 54246 | 0.001 |
| Right-Putamen | 40364 | <0.001 | 55189 | 0.002 |
| Left-Pallidum | 41740 | <0.001 | 55460 | 0.003 |
| Right-Pallidum | 58999 | 0.064 | 59580 | 0.094 |
| Left-Hippocampus | 53470 | <0.001 | 51441 | <0.001 |
| Right-Hippocampus | 50042 | <0.001 | 53284 | <0.001 |
| Left-Amygdala | 31851 | <0.001 | 53636 | <0.001 |
| Right-Amygdala | 43708 | <0.001 | 52758 | <0.001 |
| Left-Accumbens-area | 43967 | <0.001 | 55662 | 0.004 |
| Right-Accumbens-area | 40793 | <0.001 | 50969 | <0.001 |
| eTIV | 56894 | 0.013 | 64150 | 0.763 |

LH, left; RH, right; Vis, visual; SomMot, somatomotor; DorsAttn, dorsal attention; SalVenAttn, ventral attention; Cont, control; PrCv, precentral ventral; FEF, frontal eye fields, ParOper, parietal operculum; FrOper, frontal operculum; Ins, insula; PFCl, lateral prefrontal cortex; OFC, orbitofrontal cortex, TempPole, temporal; pCun, precuneus; Cing, cingulate

**Supplementary Table 5.** Clinical associations of brain-PAD

|  | Healthy control | | Recent-onset SCZ | | Chronic SCZ | |
| --- | --- | --- | --- | --- | --- | --- |
|  | beta | uncorrected p | beta | uncorrected p | beta | uncorrected p |
| FSIQ | -0.0555 | 0.273 | -0.0079 | 0.749 | -0.0402 | 0.042 |
| MQ | 0.0422 | 0.266 | -0.0215 | 0.274 | -0.0128 | 0.480 |
| PANSS positive |  |  | -0.0226 | 0.655 | -0.0634 | 0.282 |
| PANSS negative |  |  | 0.0259 | 0.638 | -0.0677 | 0.117 |
| PANSS general |  |  | -0.0170 | 0.736 | -0.0413 | 0.126 |
| PANSS total |  |  | -0.0040 | 0.867 | -0.0224 | 0.114 |
| GAF |  |  | -0.0313 | 0.403 | 0.0339 | 0.170 |

FSIQ, full-scale intelligence quotient; MQ, memory quotient; PAD, predicted age difference; PANSS, positive and negative syndrome scale; GAF, global assessment of functioning; SCZ, schizophrenia

**Supplementary Table 6.** Between-group differences in the SHAP value and group by SHAP value interactions

|  | Group | | Group X SHAP value | |
| --- | --- | --- | --- | --- |
| Feature | F | FDR p | F | FDR p |
| eTIV | 0.743 | 0.636 | 1.02 | 0.518 |
| LH Putamen | 1.228 | 0.611 | 3.889 | 0.110 |
| LH SomMot_3_surface | 0.605 | 0.668 | 0.884 | 0.518 |
| LH Default_PFC_5_thickness | 4.424 | 0.132 | 0.891 | 0.518 |
| RH Pallidum | 0.766 | 0.636 | 1.388 | 0.387 |
| RH Putamen | 1.81 | 0.554 | 2.284 | 0.190 |
| LH SalVentAttn_FrOperIns_1_surface | 0.924 | 0.613 | 3.399 | 0.111 |
| LH Vis_6_thickness | 0.002 | 0.998 | 4.386 | 0.091 |
| LH SomMot_2_surface | 1.193 | 0.611 | 0.821 | 0.519 |
| RH Cont_PFCl_1_thickness | 7.062 | 0.022 | 2.779 | 0.129 |
| RH Accumbens area | 1.058 | 0.613 | 0.62 | 0.599 |
| LH Limbic_OFC_1_surface | 1.409 | 0.611 | 3.088 | 0.115 |
| RH Default_PFCdPFCm_2_thickness | 3.483 | 0.195 | 3.431 | 0.111 |
| RH SomMot_2_surface | 0.135 | 0.971 | 3.001 | 0.115 |
| LH SalVentAttn_Med_3_thickness | 2.491 | 0.341 | 2.136 | 0.201 |
| LH Default_PFC_3_thickness | 3.295 | 0.195 | 6.198 | 0.049 |
| RH Thalamus | 1.654 | 0.554 | 0.089 | 0.915 |
| RH Vis_4_thickness | 0.568 | 0.668 | 0.385 | 0.716 |
| RH SomMot_5_surface | 0.950 | 0.613 | 3.297 | 0.111 |
| LH DorsAttn_Post_1_thickness | 0.038 | 0.998 | 5.429 | 0.051 |

SHAP, SHapley Additive exPlanations; FDR, false-discovery rate; eTIV, estimated total intracranial volume; LH, left; RH, right; SomMot, somatomotor; PFC, prefrontal cortex; SalVenAttn, salience/ ventral attention; FrOperIns, frontal-operculum-insula; Vis, visual; Cont, control; PFCl, lateral prefrontal cortex; OFC, orbitofrontal cortex; PFCdPFCm, dorsal-medial prefrontal cortex; Med, medial; DorsAttn, dorsal attention


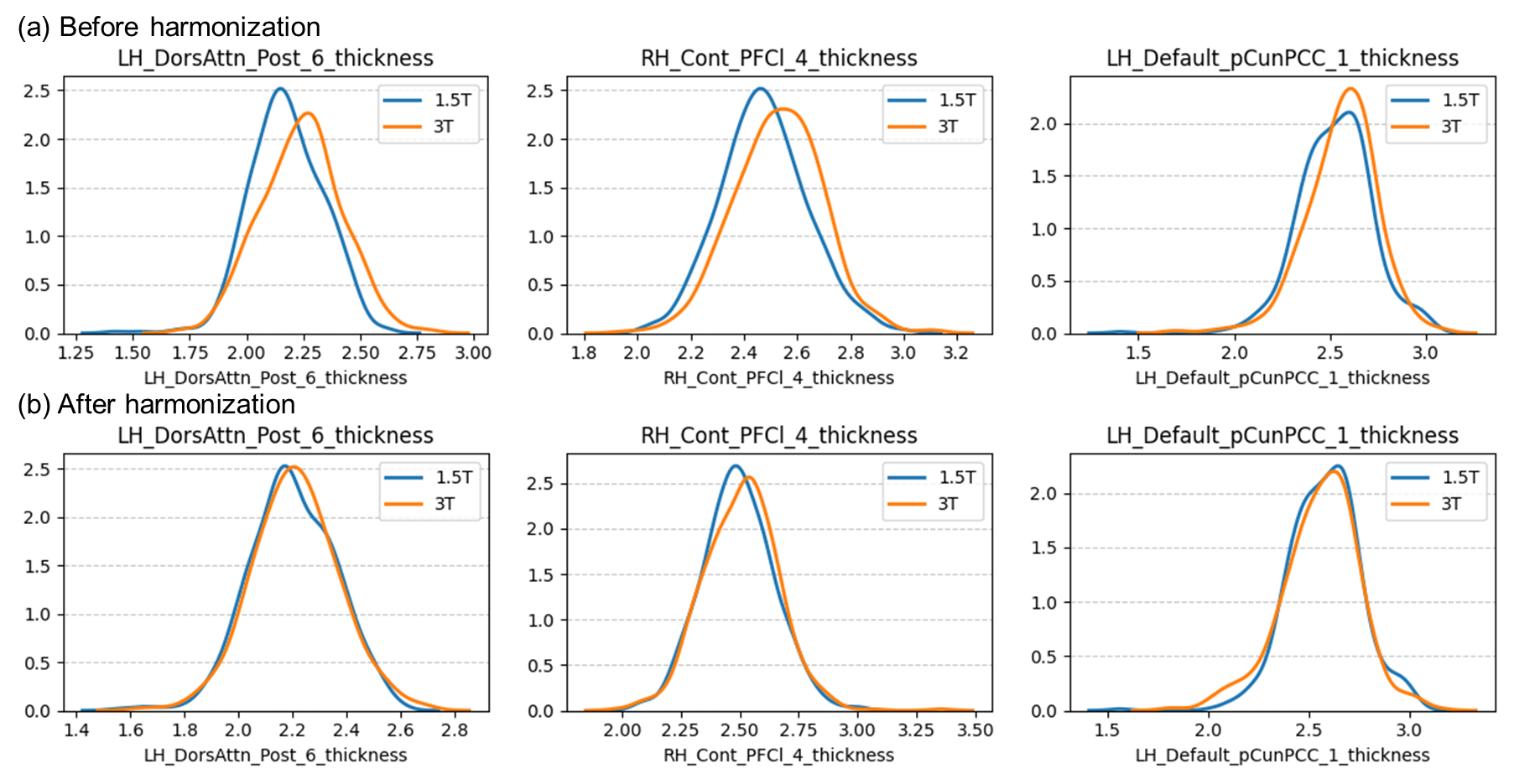


**Supplementary Figure 1. Distributions of cortical thickness in three representative regions (post central cortex, lateral prefrontal cortex, and precuneus posterior cingulate cortex) for 1.5T and 3T scanner.** (a) before harmonization and (b) after harmonization.


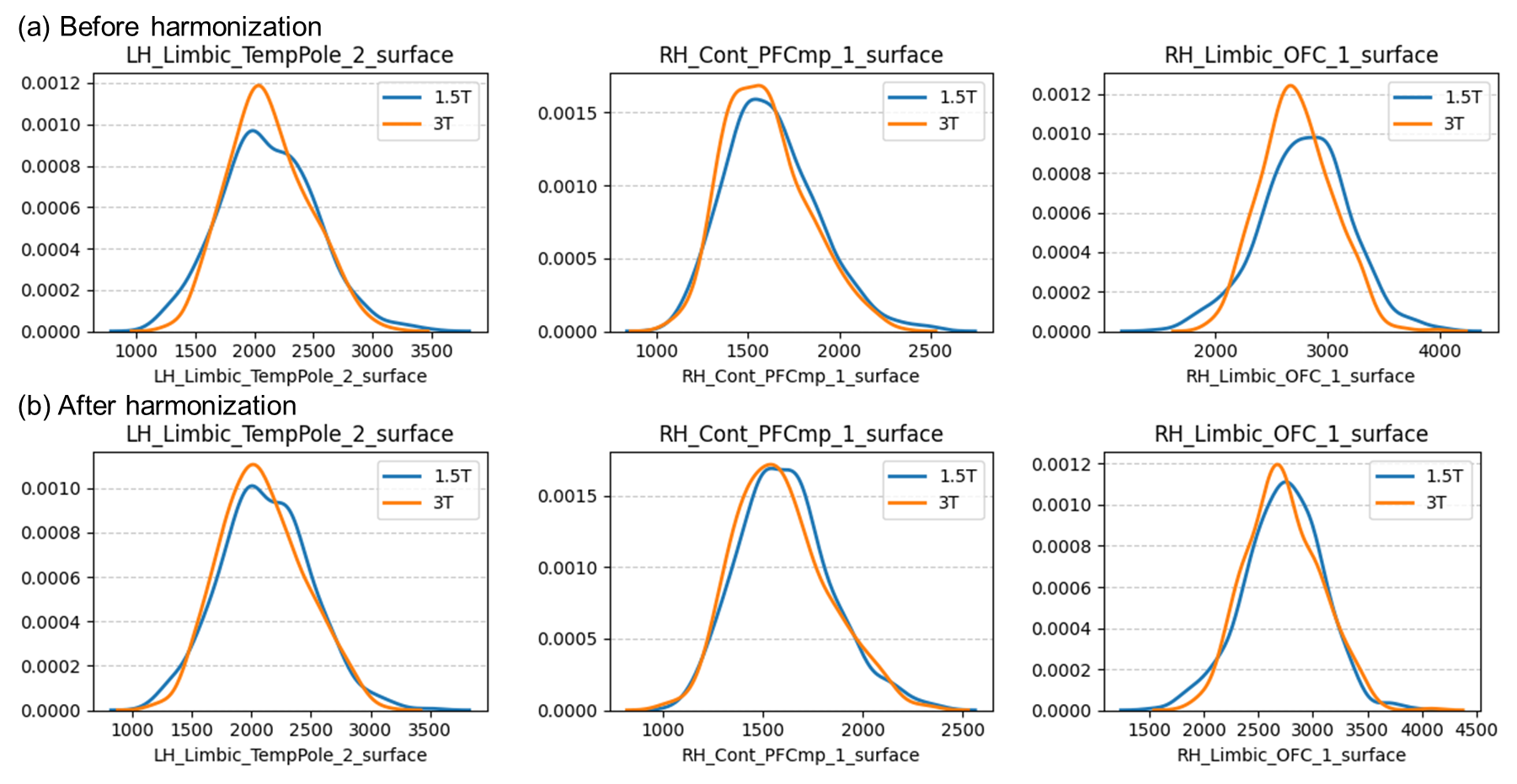


**Supplementary Figure 2. Distributions of surface area in three representative regions (temporal pole, medial posterior prefrontal cortex, and orbital frontal cortex) for 1.5T and 3T scanner.** (a) before harmonization and (b) after harmonization.


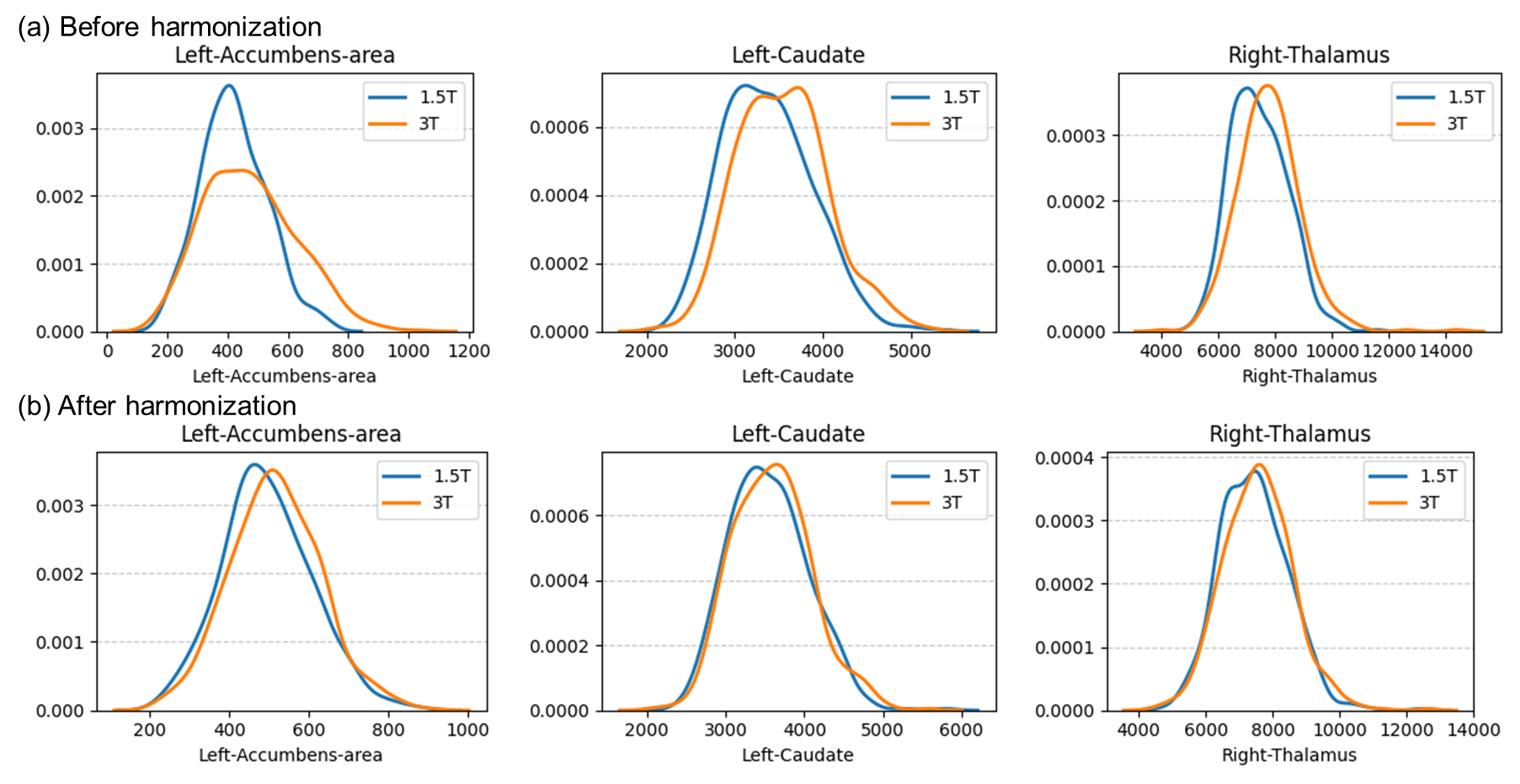


**Supplementary Figure 3. Distributions of subcortical volume in three representative regions (accumbens, caudate, and thalamus) for 1.5T and 3T scanner.** (a) before harmonization and (b) after harmonization.


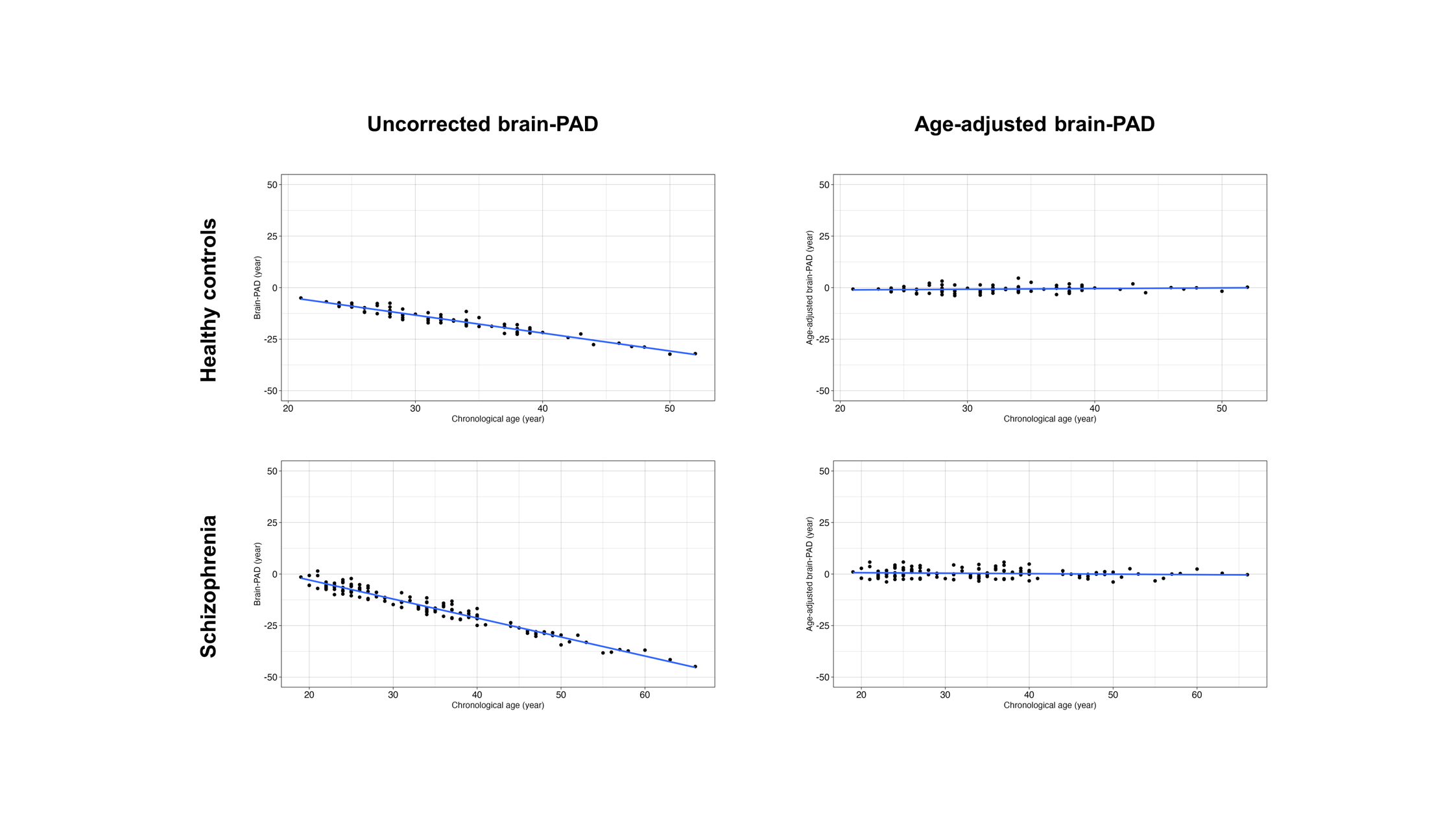


**Supplementary Figure 4. Associations between chronological age and brain-predicted age difference (brain-PAD) in healthy controls and patients with schizophrenia.** The left panel demonstrates a strong negative correlation between chronological age and brain-PAD in both groups. The right panel shows that age-adjusted brain-PAD was successfully corrected for age-related deviations.

**
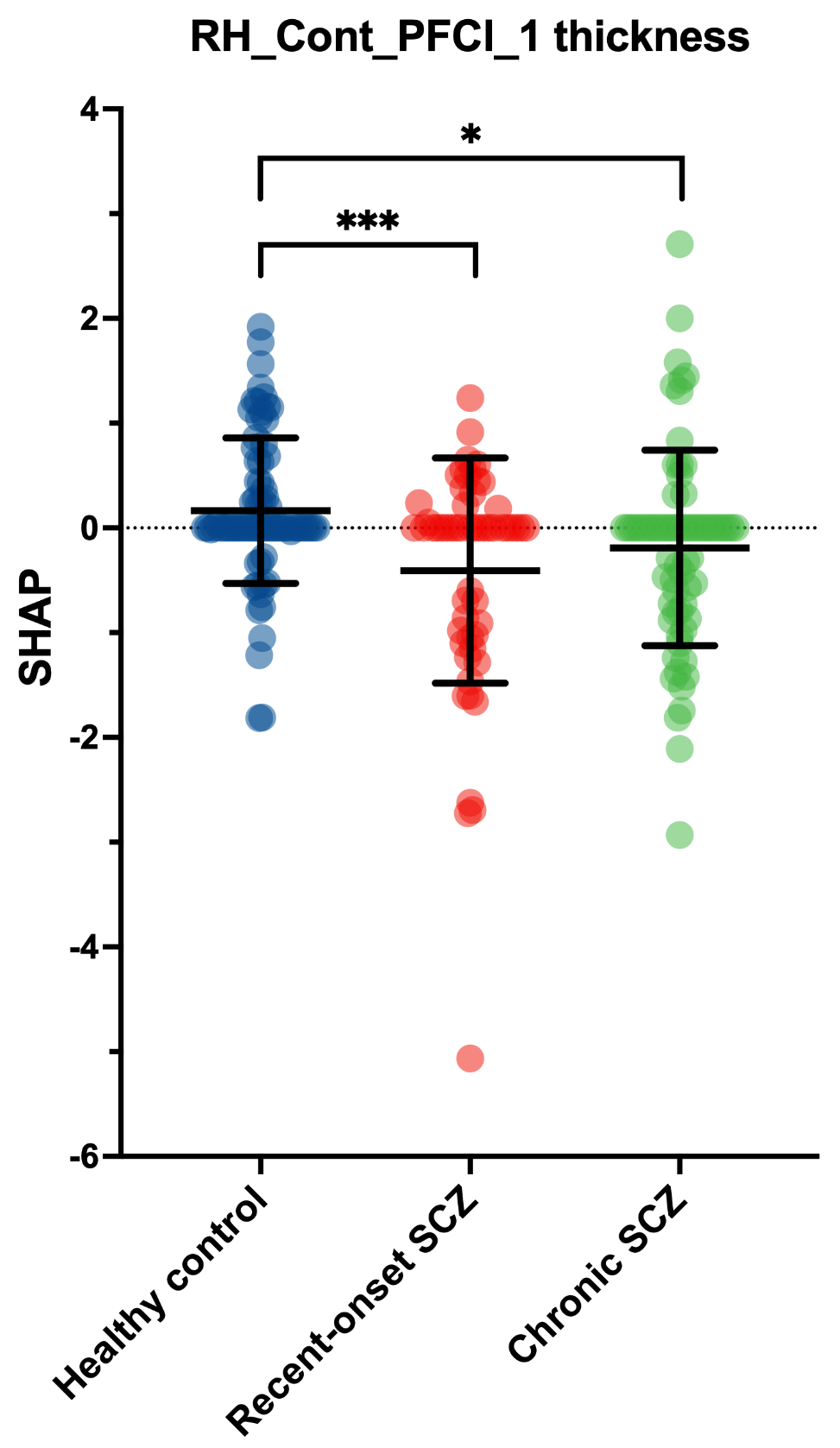
**

**Supplementary Figure 5. Differences in SHAP values across the groups.** Blue, red, and green dots represent healthy control, patients with recent-onset schizophrenia, and patients with chronic schizophrenia, respectively. Black lines represent means and standard deviations of the SHAP values in each group. SHAP, SHapley Additive exPlanations; SCZ, schizophrenia. * p<0.05, *** p<0.001.

**Reference**

Aine, C., H. J. Bockholt, J. R. Bustillo, J. M. Cañive, A. Caprihan, C. Gasparovic, F. M. Hanlon, J. M. Houck, R. E. Jung and J. Lauriello (2017). "Multimodal neuroimaging in schizophrenia: description and dissemination." Neuroinformatics **15**: 343-364.

Alexander, L. M., J. Escalera, L. Ai, C. Andreotti, K. Febre, A. Mangone, N. Vega-Potler, N. Langer, A. Alexander and M. Kovacs (2017). "An open resource for transdiagnostic research in pediatric mental health and learning disorders." Scientific data **4**(1): 1-26.

Biswal, B. B., M. Mennes, X.-N. Zuo, S. Gohel, C. Kelly, S. M. Smith, C. F. Beckmann, J. S. Adelstein, R. L. Buckner and S. Colcombe (2010). "Toward discovery science of human brain function." Proceedings of the national academy of sciences **107**(10): 4734-4739.

Botvinik-Nezer, R., R. Iwanir, F. Holzmeister, J. Huber, M. Johannesson, M. Kirchler, A. Dreber, C. F. Camerer, R. A. Poldrack and T. Schonberg (2019). "fMRI data of mixed gambles from the Neuroimaging Analysis Replication and Prediction Study." Scientific data **6**(1): 106.

Breiman, L. (1996). "Bagging predictors." Machine learning **24**: 123-140.

Breiman, L. (2001). "Random forests." Machine learning **45**: 5-32.

Chen, T. and C. Guestrin (2016). Xgboost: A scalable tree boosting system. Proceedings of the 22nd acm sigkdd international conference on knowledge discovery and data mining.

Cover, T. and P. Hart (1967). "Nearest neighbor pattern classification." IEEE transactions on information theory **13**(1): 21-27.

Dudani, S. A. (1976). "The distance-weighted k-nearest-neighbor rule." IEEE Transactions on Systems, Man, and Cybernetics(4): 325-327.

Erickson, N., J. Mueller, A. Shirkov, H. Zhang, P. Larroy, M. Li and A. Smola (2020). "Autogluon-tabular: Robust and accurate automl for structured data." arXiv preprint arXiv:2003.06505.

Geurts, P., D. Ernst and L. Wehenkel (2006). "Extremely randomized trees." Machine learning **63**: 3-42.

Gollub, R. L., J. M. Shoemaker, M. D. King, T. White, S. Ehrlich, S. R. Sponheim, V. P. Clark, J. A. Turner, B. A. Mueller and V. Magnotta (2013). "The MCIC collection: a shared repository of multi-modal, multi-site brain image data from a clinical investigation of schizophrenia." Neuroinformatics **11**: 367-388.

Holmes, A. J., M. O. Hollinshead, T. M. O’keefe, V. I. Petrov, G. R. Fariello, L. L. Wald, B. Fischl, B. R. Rosen, R. W. Mair and J. L. Roffman (2015). "Brain Genomics Superstruct Project initial data release with structural, functional, and behavioral measures." Scientific data **2**(1): 1-16.

Howard, J. and S. Gugger (2020). "Fastai: a layered API for deep learning." Information **11**(2): 108.

Ke, G., Q. Meng, T. Finley, T. Wang, W. Chen, W. Ma, Q. Ye and T.-Y. Liu (2017). "Lightgbm: A highly efficient gradient boosting decision tree." Advances in neural information processing systems **30**.

Kogan, A., K. Alpert, J. L. Ambite, D. S. Marcus and L. Wang (2016). "Northwestern University schizophrenia data sharing for SchizConnect: A longitudinal dataset for large-scale integration." Neuroimage **124**: 1196-1201.

LaMontagne, P. J., T. L. Benzinger, J. C. Morris, S. Keefe, R. Hornbeck, C. Xiong, E. Grant, J. Hassenstab, K. Moulder and A. G. Vlassenko (2019). "OASIS-3: longitudinal neuroimaging, clinical, and cognitive dataset for normal aging and Alzheimer disease." medrxiv: 2019.2012. 2013.19014902.

Liu, W., D. Wei, Q. Chen, W. Yang, J. Meng, G. Wu, T. Bi, Q. Zhang, X.-N. Zuo and J. Qiu (2017). "Longitudinal test-retest neuroimaging data from healthy young adults in southwest China." Scientific data **4**(1): 1-9.

Park, J., J. Carp, K. M. Kennedy, K. M. Rodrigue, G. N. Bischof, C.-M. Huang, J. R. Rieck, T. A. Polk and D. C. Park (2012). "Neural broadening or neural attenuation? Investigating age-related dedifferentiation in the face network in a large lifespan sample." Journal of Neuroscience **32**(6): 2154-2158.

Poldrack, R. A., E. Congdon, W. Triplett, K. Gorgolewski, K. Karlsgodt, J. Mumford, F. Sabb, N. Freimer, E. London and T. Cannon (2016). "A phenome-wide examination of neural and cognitive function." Scientific data **3**(1): 1-12.

Prokhorenkova, L., G. Gusev, A. Vorobev, A. V. Dorogush and A. Gulin (2018). "CatBoost: unbiased boosting with categorical features." Advances in neural information processing systems **31**.

Shafto, M. A., L. K. Tyler, M. Dixon, J. R. Taylor, J. B. Rowe, R. Cusack, A. J. Calder, W. D. Marslen-Wilson, J. Duncan and T. Dalgleish (2014). "The Cambridge Centre for Ageing and Neuroscience (Cam-CAN) study protocol: a cross-sectional, lifespan, multidisciplinary examination of healthy cognitive ageing." BMC neurology **14**: 1-25.

Snoek, L., M. M. van der Miesen, T. Beemsterboer, A. Van Der Leij, A. Eigenhuis and H. Steven Scholte (2021). "The Amsterdam Open MRI Collection, a set of multimodal MRI datasets for individual difference analyses." Scientific data **8**(1): 85.

Sunavsky, A. and J. Poppenk (2020). "Neuroimaging predictors of creativity in healthy adults." Neuroimage **206**: 116292.

Tanaka, S. C., A. Yamashita, N. Yahata, T. Itahashi, G. Lisi, T. Yamada, N. Ichikawa, M. Takamura, Y. Yoshihara and A. Kunimatsu (2021). "A multi-site, multi-disorder resting-state magnetic resonance image database." Scientific data **8**(1): 227.

Tian, L., J. Wang, C. Yan and Y. He (2011). "Hemisphere-and gender-related differences in small-world brain networks: a resting-state functional MRI study." Neuroimage **54**(1): 191-202.

Van Essen, D. C., S. M. Smith, D. M. Barch, T. E. Behrens, E. Yacoub, K. Ugurbil and W.-M. H. Consortium (2013). "The WU-Minn human connectome project: an overview." Neuroimage **80**: 62-79.

Wei, D., K. Zhuang, L. Ai, Q. Chen, W. Yang, W. Liu, K. Wang, J. Sun and J. Qiu (2018). "Structural and functional brain scans from the cross-sectional Southwest University adult lifespan dataset." Scientific data **5**(1): 1-10.

Wolpert, D. H. (1992). "Stacked generalization." Neural networks **5**(2): 241-259.

Zuo, X.-N., J. S. Anderson, P. Bellec, R. M. Birn, B. B. Biswal, J. Blautzik, J. Breitner, R. L. Buckner, V. D. Calhoun and F. X. Castellanos (2014). "An open science resource for establishing reliability and reproducibility in functional connectomics." Scientific data **1**(1): 1-13.
